# Positive feedback regulation between glycolysis and histone lactylation drives oncogenesis in pancreatic ductal adenocarcinoma

**DOI:** 10.1186/s12943-024-02008-9

**Published:** 2024-05-06

**Authors:** Fei Li, Wenzhe Si, Li Xia, Deshan Yin, Tianjiao Wei, Ming Tao, Xiaona Cui, Jin Yang, Tianpei Hong, Rui Wei

**Affiliations:** 1 Department of Endocrinology and Metabolism, State Key Laboratory of Female Fertility Promotion, Peking University Third Hospital, Beijing, 100191 China; 2 Department of Laboratory Medicine, Peking University Third Hospital, Beijing, 100191 China; 3 Department of General Surgery, Peking University Third Hospital, Beijing, 100191 China

**Keywords:** Glycolysis, Histone lactylation, H3K18la, Pancreatic ductal adenocarcinoma

## Abstract

**Background:**

Metabolic reprogramming and epigenetic alterations contribute to the aggressiveness of pancreatic ductal adenocarcinoma (PDAC). Lactate-dependent histone modification is a new type of histone mark, which links glycolysis metabolite to the epigenetic process of lactylation. However, the role of histone lactylation in PDAC remains unclear.

**Methods:**

The level of histone lactylation in PDAC was identified by western blot and immunohistochemistry, and its relationship with the overall survival was evaluated using a Kaplan-Meier survival plot. The participation of histone lactylation in the growth and progression of PDAC was confirmed through inhibition of histone lactylation by glycolysis inhibitors or lactate dehydrogenase A (*LDHA*) knockdown both in vitro and in vivo. The potential writers and erasers of histone lactylation in PDAC were identified by western blot and functional experiments. The potential target genes of H3K18 lactylation (H3K18la) were screened by CUT&Tag and RNA-seq analyses. The candidate target genes TTK protein kinase (*TTK*) and BUB1 mitotic checkpoint serine/threonine kinase B (*BUB1B*) were validated through ChIP-qPCR, RT-qPCR and western blot analyses. Next, the effects of these two genes in PDAC were confirmed by knockdown or overexpression. The interaction between TTK and LDHA was identified by Co-IP assay.

**Results:**

Histone lactylation, especially H3K18la level was elevated in PDAC, and the high level of H3K18la was associated with poor prognosis. The suppression of glycolytic activity by different kinds of inhibitors or *LDHA* knockdown contributed to the anti-tumor effects of PDAC in vitro and in vivo. E1A binding protein p300 (P300) and histone deacetylase 2 were the potential writer and eraser of histone lactylation in PDAC cells, respectively. H3K18la was enriched at the promoters and activated the transcription of mitotic checkpoint regulators *TTK* and *BUB1B*. Interestingly, TTK and BUB1B could elevate the expression of P300 which in turn increased glycolysis. Moreover, TTK phosphorylated LDHA at tyrosine 239 (Y239) and activated LDHA, and subsequently upregulated lactate and H3K18la levels.

**Conclusions:**

The glycolysis-H3K18la-TTK/BUB1B positive feedback loop exacerbates dysfunction in PDAC. These findings delivered a new exploration and significant inter-relationship between lactate metabolic reprogramming and epigenetic regulation, which might pave the way toward novel lactylation treatment strategies in PDAC therapy.

**Supplementary Information:**

The online version contains supplementary material available at 10.1186/s12943-024-02008-9.

## Introduction

Pancreatic cancer, of which over 80% is pancreatic ductal adenocarcinoma (PDAC), is a highly aggressive tumor of the digestive system and the most lethal human malignancy, with a poor overall 5-year survival rate [1]. Despite attempts to improve its treatment over the past decade, the mortality rates for PDAC have not considerably declined. Therefore, it is imperative for a clearer understanding of PDAC growth and metastasis mechanisms, to find potential therapeutic targets and novel biomarkers to predict PDAC prognosis [2, 3].

Besides mutations in PDAC driver genes such as oncogenic *KRAS*, tumor suppressor genes *SMAD4* and *TP53* [4], numerous studies have shown that the aggressive tumor biology of PDAC is closely related to metabolites and epigenetic rewiring [5, 6]. PDAC cells utilize “metabolic reprogramming” to support malignant behaviors such as rapid proliferation, invasion and several cellular processes [7]. Metabolites are intermediate products of cellular metabolism which are catalyzed by various enzymes, via corresponding post-translational modifications of proteins. More and more metabolites have been reported to be involved in the regulation of diverse signaling pathways in tumorigenesis [8]. Among these, lactate, an abundant oncometabolite, is an end product of glycolysis and fuels into the tricarboxylic acid cycle during the Warburg effect (aerobic glycolysis) [9, 10]. Enhanced glycolysis and accumulation of lactate are common features in various types of cancer [11]. However, how metabolic reprogramming reshapes epigenetic alterations, in particular lactylation in PDAC, is still unclear.

Histones are involved in regulating various physiological functions, and post-translational modifications of histones (such as acetylation, succinylation) play important roles in the development of diseases [12]. Lactate-derived lactylation on core histones has been confirmed by several groups as a new type of histone mark [13, 14]. Till now, 28 histone lactylation sites have been identified, including histone H3 lysine 4 and histone H3 lysine 18 (H3K18) et al. Histones exert biological effects by affecting the expression and activation of the downstream genes. Lactylation of histones preferentially affects enzymes participating in essential metabolic pathways such as carbohydrate, amino acid, lipid, and nucleotide metabolism [15]. However, strategies involving histone lactylation treatment to overcome and shift molecular landscape of PDAC have not been achieved. Thus, a better understanding of histone lactylation in the pathology of PDAC still requires further investigation.

Our study aims to investigate the role of histone lactylation, specifically H3K18la, in PDAC progression and to clarify the potential mechanism. Our results demonstrated that lactate accumulation in the PDAC tumor microenvironment drove histone lactylation, both in vivo and in vitro, correlating strongly with tumorigenesis and poor clinical outcomes. Mechanistically, enhanced glycolysis in PDAC led to increased lactate production and H3K18la, which promoted the transcription of TTK protein kinase (*TTK*) and BUB1 mitotic checkpoint serine/threonine kinase B (*BUB1B*). Notably, TTK and BUB1B upregulated P300 (the writer of histone lactylation); TTK activated lactate dehydrogenase A (LDHA), upregulated lactate production and promoted histone lactylation, thus forming a feedback loop of glycosis-H3K18la-TTK/BUB1B. Our findings, as a proof of principle, for the first time, uncover a novel role of histone lactylation especially H3K18la in PDAC, while disruption of the glycolysis-H3K18la-TTK/BUB1B positive feedback loop may be a potential therapeutic approach for the supplementary treatment of PDAC.

## Materials and methods

### Human samples

The pancreatic samples of PDAC and paired para-carcinoma controls were archived in the Department of General Surgery at Peking University Third Hospital. All tissues were immediately frozen in liquid nitrogen and stored at − 80 °C for lactate level and lactylation detection. The serum samples of PDAC and healthy controls were collected from the Department of Laboratory Medicine at Peking University Third Hospital for non-targeted metabolomics assay. All participants in this study provided written informed consent and the study was approved by the Clinical Research Ethics Committee of Peking University Third Hospital. One tissue microarray (TMA, Cat. HPanA180Su03) containing tumor and the paired para-carcinoma tissues from 90 PDAC patients was obtained from Outdo Biotech Co., Ltd (Shanghai, China), and ethical approval was granted by the Clinical Research Ethics Committee, Outdo Biotech Co., Ltd.

### Non-targeted metabolomics assay

The non-targeted metabolomics assay experiments were performed by APExBIO Technology, Llc (Shanghai, China). In a 1.5 mL centrifuge tube, 100 µL of the serum was combined with 300 µL of methanol solution containing the internal standard L-2-chlorophenylalanine 10 µL/mL. A quanlity control sample was prepared by extracting 20 µL of the supernatant from each sample, followed by thorough mixing and vortexing. Liquid chromatograph-mass spectrometer analysis was performed using an Agilent 1290 Infinity IIUHPLC system coupled to an Agilent 6545 UHD and Accurate-Mass Q-TOF/MS. The chromatographic column utilized was Waters XSelect-HSS T3. Mass spectrometry was conducted in both positive and negative ion modes with the following optimized parameters: Capillary voltage was set at 4.5 kV in positive mode and 3.5 kV in negative mode. The drying gas flow was 8 L/min in positive mode and 10 L/min in negative mode, with a gas temperature of 325℃. Nebulizer pressure was set to 20 psig. The screening criteria for differential metabolites were variable importance in projection (VIP) > 1, |log_2_Fold Change| > 1, *P* < 0.05.

### Cell culture and treatment

Human pancreatic ductal epithelial cell line hTERT-HPNE and four PDAC cell lines (MIA PaCa-2, PANC-1, AsPC-1 and PL45) were used in this study. hTERT-HPNE cells were obtained from Meisen Cell Technology Co., Ltd (Zhejiang, China). MIA PaCa-2 cells were obtained from the Chinese Academy of Sciences Cell Bank (Shanghai, China). PANC-1 and AsPC-1 cells were obtained from the Cell Resource Center, Peking Union Medical College (Beijing, China). PL45 cells were obtained from iCell Bioscience Inc. (Shanghai, China). The cells were cultured in Dulbecco’s modified Eagle’s medium (DMEM) or Roswell Park Memorial Institute (RPMI) 1640 medium supplemented with 10% (vol/vol) fetal bovine serum, 1% (vol/vol) penicillin-streptomycin, 1% (vol/vol) GlutaMAX (all from Gibco, Grand Island, NY, USA). The cells were grown in a humidified atmosphere containing 5% CO_2_ at 37 °C. hTERT-HPNE and four PDAC cell lines were employed to investigate lactate production, histone lactylation, and the expression of TTK and BUB1B.

Subsequent experiments were conducted using MIA PaCa-2 and AsPC-1 cells, which underwent the following interventions: (1) Cells were treated with several glycolysis inhibitors: sodium dichloroacetate (DCA: 0–30 mmol/L), which can inhibit pyruvate dehydrogenase kinase [16]; Oxamate (0–20 mmol/L), an inhibitor of LDHA [17]; 2-deoxy-D-glucose (2-DG, 0–10 mmol/L), serving as a mimic of D-glucose, disrupting D-glucose metabolism by inhibiting hexokinase and glucose-6-phosphate isomerase [18] or the vehicle control (phosphate buffer saline, PBS). (2) Cells were transfected with *LDHA* siRNA or scramble siRNA as negative control, combined with sodium lactate (NaLa, 10 mmol/L). (3) Cells were treated with P300 inhibitor C646 (20 µmol/L) combined with NaLa (10 mmol/L). (4) Cells were transfected with *P300* siRNA or scramble siRNA as negative control, combined with NaLa (10 mmol/L). (5) Cells were treated with various histone deacetylase (HDAC) inhibitors, including broad-spectrum HDAC inhibitors (Trichostatin A, TSA, 500 nmol/L), sirtuin inhibitor (Nicotinamide, NAM, 10 mmol/L), class I HDAC inhibitor (Tacedinaline, CI994, 5 µmol/L), IIa HDAC inhibitor (TMP195, 5 µmol/L), class IIb HDAC inhibitor (Bufexamac, 250 µmol/L), and class IV HDAC inhibitor (SIS17, 25 µmol/L). (6) Cells were transfected with *HDAC1, 2, 3* overexpression plasmids or negative control plasmids. (7) Cells were transfected with *TTK* siRNA, *BUB1B* siRNA or scramble siRNA as negative control. (8) Cells were transfected with *TTK* overexpression plasmid or negative control plasmid.

After treatment, the proteins were collected for histone lactylation detection by western blot or for LDHA activity assay. Supernatant was collected for lactate level detection. Cell proliferation was monitored by using IncuCyte S3 during the 72-h intervention or detected by colony formation assays after 1–2 weeks treatment. Cell migration was monitored by using wound healing during the 48-h intervention or detected by transwell assays after 48-h treatment. The interaction between H3K18la with *TTK* or *BUB1B* was determined by chromatin immunoprecipitation-quantitative real-time PCR (ChIP-qPCR). The mRNA and protein levels were detected by reverse transcription quantitative real-time PCR (RT-qPCR) and western blot. The interaction between TTK and LDHA was assessed by co-immunoprecipitation (Co-IP) assays.

### RNA interference and overexpression

SiRNA was dissolved to a working concentration of 50 nmol/L. MIA PaCa-2 and AsPC-1 cells underwent RNA interference using Lipofectamine™ RNAi MAX (Invitrogen, CA, USA) following the manufacturer’s instructions, and cells were harvested at 48 h after transfection. The sequences of siRNA are listed in Supplementary Table S1.

Plasmids were prepared from GeneChem (Shanghai, China). MIA PaCa-2 and AsPC-1 cells were transfected with 3 µg plasmids with Lipofectamine™ 2000 (Invitrogen) for 48 h to fulfill overexpression of the specific genes.

### Lentivirus transfection

To realize the stable expression of *LDHA* knockdown and *TTK* overexpression, recombinant lentiviruses expressing sh-*LDHA* and oe-*TTK* were prepared by Hanbio Tech (Shanghai, China). MIA PaCa-2 cells at a confluence of 30–40% were transfection with lentivirus [10 multiplicities of infection (MOI), MOI = virus titer (TU/mL) x virus volume (mL) /cell number] for 24 h, and screened with 1 µg/mL puromycin (MedChemExpress, Shanghai, China) for one week and maintained in 0.4 µg/mL puromycin. After confirmation of expression with RT-qPCR and western blot, the sh-*LDHA* and oe-*TTK* stable cell lines were used for subsequent experiments. The sequences of sh-*LDHA* are listed in Supplementary Table S1.

### Cell proliferation assay

MIA PaCa-2 and AsPC-1 cells were seeded in 96-well plates at a density of 5000 cells per well and maintained for 72 h. Cell proliferation was monitored using a long-term process live cell analysis system IncuCyte S3 (Sartorius, Göttingen, Germany). Proliferation was assessed by confluence measurements and normalized to 0 h calculated by IncuCyte S3 software. Cell images were captured at 3-h intervals from four separate regions per well. There were 3–6 repetitions in each experiment.

### Colony formation assay

MIA PaCa-2 and AsPC-1 cells were seeded into 6-well plates at a concentration of 1000 cells per well. The medium was refreshed every 3 days during colony growth, and cells were cultured for 7–14 days based on the characteristics of each cell line. Subsequently, the cells were fixed with 4% paraformaldehyde (Leagene, Beijing, China) for 20 min and stained with crystal violet (Beyotime Biotechnology) for 15 min at room temperature. The resulting colonies were then photographed and counted.

### Wound healing assay

MIA PaCa-2 and AsPC-1 cells were seeded in 96-well plates and grown to 90–100% confluence, scratching wounds were generated by the IncuCyte Wound Marker tool. The shedding of the cell mass was washed off with PBS. After changing the serum-free medium, the culture was maintained for 48 h. Photographs were captured at intervals of 3-h in the IncuCyte S3 platform. The wound areas were quantified using IncuCyte S3 image analysis software.

Sometimes, cells were seeded in 6-well plates and grown to 90–100% confluence, scratching wounds were generated using a 200 µL sterile pipette tip. The shedding of the cell mass was washed off with PBS. After changing the serum-free medium, the culture was maintained for 48 h. Photographs were captured at 0, 24 and 48 h after wound generation using a computer-assisted microscope (Leica Microsystems, Wetzlar, Germany). Subsequently, the wound areas were quantified using Image J software (National Institutes of Health, Bethesda, MD, USA).

### Transwell migration assay

The migration abilities of the MIA PaCa-2 and AsPC-1 cells were evaluated using a chamber of 8-µm transwell inserts (Corning Life Science, NY, USA). 1 × 10^5^ Cells were plated in the upper transwell chamber in 200 µL medium without fetal bovine serum, while the lower chamber was filled with 600 µL medium containing 10% fetal bovine serum as the attractant. After the 48-h incubation, the migrated cells were fixed with 4% paraformaldehyde and stained with crystal violet. The stained cells were photographed, and the number of migrated cells was quantified using Image J software.

### Transplanted tumor model in nude mice

Female nude mice, aged 4–5 weeks and weighing 18–20 g, were randomly assigned to different experimental groups. (1) A total of 5 × 10^6^ MIA PaCa-2 cells were injected into each mouse subcutaneously. When the average tumor volume reached approximately 70 mm^3^, the mice were randomly assigned to the Oxamate group (*n* = 7) and control group (*n* = 7), and the tumor volumes were measured. Oxamate (750 mg/kg, daily) or vehicle (PBS) was administered by intraperitoneal injection for 30 days. (2) The stable sh-*LDHA* or negative control (sh-*NC*) MIA PaCa-2 cells (5 × 10^6^ cells per mouse) were injected into mice subcutaneously, with 7 mice in each group. When the tumor volume in the sh-*NC* group reached approximately 70 mm^3^, the tumor volumes were measured every other day for 26 days. The tumor volumes were estimated using the following formula: volume (mm^3^) = length (mm) × width (mm)^2^/2. Mice were sacrificed and the tumors were photographed and weighed. The lactate content in the tumors was detected. The H3K18la level was detected by western blot and immunohistochemistry (IHC). Cell proliferation was determined by IHC for Ki-67 staining. Liver hematoxylin and eosin (HE) staining was performed to evaluate the tumor metastasis. All animal experiments were conducted at Peking University Health Science Center and approved by Peking University Animal Care and Use Committee.

### IHC

The tissues from patients and nude mice were fixed with 10% (vol/vol) neutral-buffered formalin overnight and embedded in paraffin, and 5-µm-thick sections were prepared. Tissue sections were deparaffinized, followed by rehydrated in xylene and graded concentrations of ethanol. Quenching of endogenous peroxidase activity was achieved using 3% hydrogen peroxide, while antigen retrieval was performed with an EDTA buffer. Then, sections were incubated overnight at 4°C in a humid chamber with the indicated primary antibodies. The 3, 3’-diaminobenzidine (ZSGB-BIO, Beijing, China) detection system was employed to visualize the staining after 1-h incubation with secondary antibodies at room temperature. The antibodies used in this study are listed in Supplementary Table S2.

For the tissue microarray, IHC scores for H3K18la expression were obtained by multiplying the staining intensity with the percentage of positive cells. For other IHC staining, the mean optical density was employed to calculate the relative expression of H3K18la, Ki-67, TTK and BUB1B using Image J software.

### RNA extraction and RT-qPCR

Total RNA was extracted using Trizol (Thermo Fisher Scientific, Waltham, USA) and reversely transcribed to cDNA with a RevertAid First Strand cDNA Synthesis Kit (Thermo Fisher Scientific). The cDNA was subjected to real-time qPCR analysis with the Thunderbird SYBR qPCR Mix (TOYOBO Co., Ltd, Osaka, Japan) on a QuantStudio5 Real-Time PCR System (Thermo Fisher Scientific). The primer sequences synthesized by the Beijing Tsingke Biotech Co., Ltd. (Beijing, China) are summarized in Supplementary Table S3. Relative gene expression was normalized to *ACTB* and calculated with the 2^−ΔΔCt^ method.

### Protein extraction and western blot

The cells or tissues were lysed using RIPA lysis buffer (Beyotime Biotechnology, Shanghai, China), which contained protease inhibitor and phosphatase inhibitor (Applygen Technologies, Beijing, China). Total protein content was quantified using the bicinchoninic acid (BCA) assay (Thermo Fisher Scientific). The denatured proteins were separated by SDS-PAGE and transferred onto a nitrocellulose membrane (Millipore, Saint Charles, MO, USA). After blocking with 5% bovine serum albumin for 1 h at room temperature, the membranes were then incubated with primary antibodies overnight at 4 °C, followed by three washes, and subsequent incubation with secondary antibodies for 1 h at room temperature. Protein bands were visualized using the Odyssey 290 infrared imaging system (LI-COR Biosciences, Lincoln, NE, USA). The intensity of each band was analyzed using Image J software. Relative protein level was normalized to β-actin. The antibodies used in this experiment are listed in Supplementary Table S2.

### Lactate content and LDH enzyme activity measurement

The supernatant of homogenized tumor tissue or the supernatant of cell culture was collected after 12,000 *g* centrifugation for 10 min. The production of lactate was measured according to the instructions of the lactic acid assay kit (Solarbio, Beijing, China). The absorbance values at 570 nm were measured using a spectrophotometer. The content of lactate was normalized based on the cell number.

LDH enzyme activity was measured by a lactate dehydrogenase assay kit (Nanjing Jiancheng Bioengineering Institute, Nanjing, China) as the manufacturer’s instructions. Briefly, cells were harvested, subjected to ultrasonication, and subsequently centrifuged at 4000 rpm for 10 min. The resulting supernatant was then collected for subsequent measurements. Absorbance at 450 nm was measured using a spectrophotometer, and the obtained results were normalized based on the total protein content.

### Co-IP

1 × 10^7^ MIA PaCa-2 and AsPC-1 cells were collected and lysed in IP lysis buffer in the presence of protease inhibitors for 40 min, and centrifuged at 4 °C, 14,000 *g* for 10 min. Lysates were divided into input, IgG and LDHA groups. The lysates were incubated with LDHA antibody or IgG at 4 °C overnight. Protein A/G magnetic beads (Beyotime) were added to lysates and incubated for 4 h at 4 °C. Following three washes with IP lysis buffer, the coprecipitated proteins were obtained and subjected to analysis through western blot.

### CUT&Tag assay

The CUT&Tag experiment was performed by Wuhan Frasergen Bioinformatics Co., Ltd (Wuhan, China). Briefly, 2 × 10^6^ cells were harvested and washed. Concanavalin A-coated magnetic beads were added and incubated for 10 min. Cells bound to the beads were then resuspended in dig-wash buffer with the H3K18la antibody for 2 h. Then cells were washed twice using the magnet stand with dig-wash buffer, and incubated with pG-Tn5 at room temperature for 1 h. After two washes, cells were resuspended in tagmentation buffer and incubated at 37 °C for 1 h. To halt tagmentation, 20 mg/mL Proteinase K, 10% SDS and 0.5 mol/L EDTA were introduced to the sample. This mixture was then incubated at 55 °C for 1 h, followed by purification using phenol–chloroform–isoamyl alcohol, ethanol precipitation, washing with 100% ethanol, and final suspension in water. DNA was amplified through PCR, and purified. The libraries were then sequenced on the Illumina Nova-seq platform. DiffBind was employed to detect differential peaks (DP) from peak sets. Peaks with |log_2_Fold Change| > 0.58 and false discovery rate (FDR) < 0.05 were defined as the statistically significant DP. The DP signal of samples was clustered for analysis. Subsequently, ChIPseeker was utilized to annotate the DP to the promoter region and to find the genes related with DP. Furthermore, the loss of DP in promoter region-associated genes were used to perform Gene Ontology (GO) and Kyoto Encyclopedia of Genes and Genomes (KEGG) analyses.

### RNA-seq

The RNA-seq experiment was performed by Wuhan Frasergen Bioinformatics Co., Ltd. Total RNA extraction was carried out using Trizol (Invitrogen). RNA purity and integrity were assessed using a NanoDrop 2000 spectrophotometer (NanoDrop Technologies, Wilmington, DE, USA). 1.5% Agarose gel was used to assess the RNA contamination. Oligo (dT)-attached magnetic beads were employed to purify mRNA. The purified mRNA was subsequently fragmented into small pieces using a fragment buffer. First-strand cDNA was synthesized through random hexamer-primed reverse transcription, followed by a second-strand cDNA synthesis, and purified using AMPure XP Beads. Following that, RNA Index Adapters and A-Tailing Mix were added through an incubation process to complete cDNA repair. The cDNA fragments underwent PCR amplification, and the resulting products were purified to obtain the final library. Once the library was tested and deemed of sufficient quality, DNA Nano Ball was prepared and subsequently loaded onto the sequencing chip. The sequencing process was then carried out using the MGI high-throughput sequencer. The screening criteria for identifying differential expressed genes included |log_2_Fold Change| > 0.58, *P* < 0.01. Subsequently, the downregulated genes were subjected to GO and KEGG analyses.

### ChIP assay and ChIP-qPCR

MIA PaCa-2 cells were harvested and fixed using 1% formaldehyde in PBS for 10 min. Then, 1.25 mol/L glycine was added to stop the crosslinking process. Following the washing step, the fixed cells were resuspended in lysis buffer (1% SDS, 50 mmol/L Tris-HCl pH 8.1, 5 mmol/L EDTA, protease inhibitors) for 1 h. To produce chromatin fragments approximately 300 bp in length, cells were subjected to sonication and centrifuge at 4 °C, 4000 rpm for 10 min. The lysates were diluted in a buffer containing 1% Triton-X-100, 20 mmol/L Tris-HCl (pH 8.1), 150 mmol/L NaCl, 2 mmol/L EDTA and protease inhibitors. Rabbit anti-IgG (2 µg) or rabbit anti-H3K18la (2 µg) was introduced into the diluted chromatin and left overnight at 4 °C with constant rotation. The following day, 30 µL of Dynabeads™ protein G (Invitrogen) were added, and incubated for 4 h at 4 °C. Following washing steps, input and the pulled-down chromatin complex were de-crosslinked at 65 °C for 12 h. The DNA obtained from the pull-down was then purified using the MinElute PCR purification kit (Qiagen, Hilden, Germany). ChIP-qPCR was performed using PerfectStart Green qPCR SuperMix (TransGen Biotech, Beijing, China) on a QuantStudio5 Real-Time PCR System. The primer sequences for ChIP-qPCR are listed in Supplementary Table S4.

### Statistical analysis

GEPIA and GEO database were utilized to analyze the expression levels of TTK and BUB1B in PDAC patients and healthy controls. GEPIA and Kaplan-Meier plotter database were used to assess the relationship between TTK/BUB1B expression and survival outcomes in PDAC patients. Data are expressed as the mean ± SD or median with interquartile range. Normality was checked by using Shapiro-Wilk test. If data were normally distributed, the differences between two groups were performed by Student’s *t*-test (two-tailed); the differences among multiple groups were performed by ANOVA, followed by post hoc Tukey’s multiple comparisons test, Dunnett’s multiple comparisons test, as appropriate. If data were not normally distributed, the differences between two groups were performed by Mann-Whitney test; the differences among multiple groups were performed by Kruskal-Wallis test followed by Dunn’s multiple comparisons test between groups.* P* < 0.05 was considered as statistically significant. Statistical analysis was conducted with GraphPad Prism 8.0 (GraphPad Software Inc., San Diego, CA).

## Results

### Elevated histone lactylation levels are associated with unfavorable prognosis in patients with PDAC

**Fig. 1:**
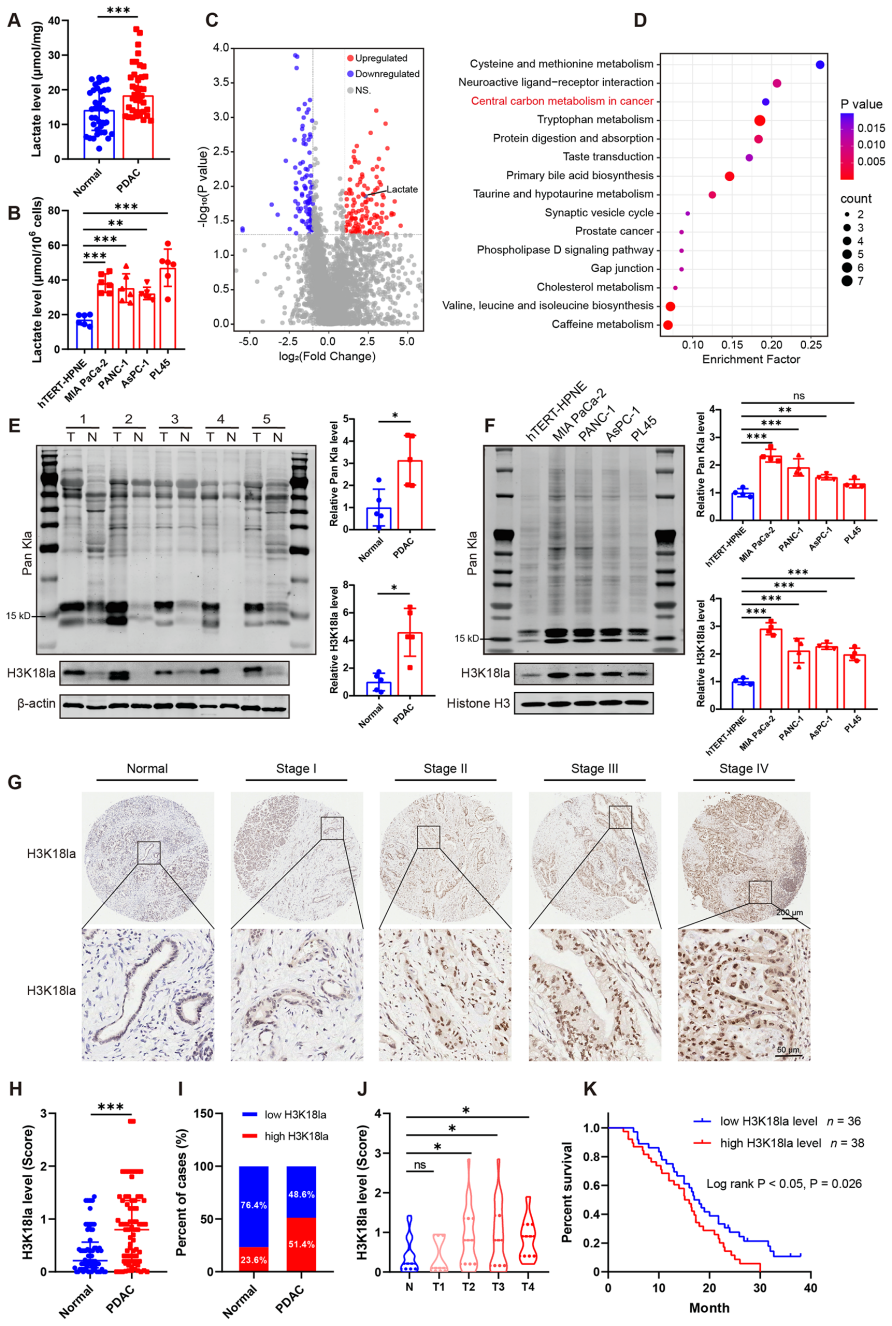
Elevated lactate level and histone lactylation are associated with unfavorable prognosis in patients with PDAC. (**A**) Lactate content in pancreatic ductal adenocarcinoma (PDAC) and paired para-carcinoma tissues. *n* = 38. (**B**) Lactate content in human pancreatic ductal epithelial cell line hTERT-HPNE and four different PDAC cell lines (MIA PaCa-2, PANC-1, AsPC-1 and PL45). *n* = 6. (**C-D**) Distinct serum metabolites in PDAC patients and healthy individuals were analyzed by using non-targeted metabolomics. *n* = 60. The volcano plot (**C**) and the KEGG analysis (**D**) on differential serum metabolites between PDAC patients and healthy individuals. (**E**) The pan-lysine lactylation (Pan Kla) and H3K18 lactylation (H3K18la) levels in paired PDAC tissue (T) and adjacent normal tissues (N) were measured by western blot. *n* = 5. Representative images (left panel) and the quantification (right panel). (**F**) The levels of Pan Kla and H3K18la in pancreatic ductal epithelial cell line and PDAC cell lines were assessed using western blot. *n* = 4. Representative images (left panel) and the quantification (right panel). (**G**-**K**) The H3K18la level in PDAC and para-carcinoma tissues were visualized by IHC staining in tissue microarray. (**G**) The representative picture of IHC staining. The visual field in the black square is enlarged below. (**H**-**I**) The quantification of IHC staining in para-carcinoma (*n* = 72) and PDAC (*n* = 74) tissues. (**J**) H3K18la levels of AJCC stages T1 to T4 in PDAC patients. There are 5 PDAC patients in T1 stage, 33 in T2 stage, 25 in T3 stage, and 11 in T4 stage. (**K**) Kaplan-Meier analysis of overall survival in PDAC patients with low (*n* = 36) and high H3K18la (*n* = 38) levels. Data in Fig. B, E, F are presented as mean ± SD; data in Fig. A, H and J are presented as median with interquartile range. Statistical analysis was performed by Student’s *t*-test in E, or by one-way ANOVA followed by Dunnett’s multiple comparisons test in B and F, or by Log-rank test in K, or by Mann-Whitney test in A and H, or by Kruskal-Wallis test followed by Dunn’s multiple comparisons in J. ^*^P < 0.05, ^**^P < 0.01, ^***^P < 0.001; ns, not significant

In pancreatic tissues from PDAC patients, the lactate content was higher than that in the normal adjacent tissues (Fig. 1A). Similarly, lactate production in PDAC cell lines such as MIA PaCa-2, PANC-1, AsPC-1 and PL45 was also higher than that in the human pancreatic ductal epithelial cell line hTERT-HPNE (Fig. 1B). Alterations of metabolites were analyzed using non-targeted metabolomics in serum samples from PDAC patients and healthy groups (the original data is in Additional file 2). Results showed that 222 metabolites differed significantly between the tumor and healthy groups, with 100 lower and 122 higher in the tumor groups, Fig. 1C). KEGG analysis of the different serum metabolites revealed enrichment in the central carbon metabolism (Fig. 1D). Especially, to our attention, lactate was also one of the metabolites.

Since the production of large amounts of lactate supplied as substrates for histone lactylation, we examined the protein lactylation levels in 5 pairs PDAC tissues and surrounding noncancerous tissues. Higher levels of global/pan-lysine lactylation (Pan Kla) were observed in PDAC tissues (Fig. 1E). As histone modifications play fundamental roles in most biological processes and H3K18la has been reported to regulate multiple biological processes such as oncogenesis [12, 19], we focused on its expression and function. Consistently, H3K18la levels were also elevated in the PDAC tissues (Fig. 1E). In addition, the expression of Pan Kla as well as H3K18la was significantly upregulated in four PDAC cell lines compared with the normal pancreatic ductal epithelial cell line (Fig. 1F). Next, we assessed the clinical importance of H3K18la by IHC staining in 74 cases of PDAC tissues and 72 cases of para-carcinoma tissue (90 paired tissues excluded the shedding slice, the tissues without pancreatic duct, the para-carcinoma tissue with obvious abnormal morphology). As shown in Fig. 1G and H, the level of H3K18la was significantly upregulated in PDAC compared with the normal tissues. Among PDAC patients, 51.4% of samples showed high H3K18la level, which were prevalent than the normal groups (Fig. 1I). The increased H3K18la levels were positively correlated with more advanced AJCC stages (Fig. 1J). To further identify the prognostic value of H3K18la, the overall survival was evaluated using a Kaplan-Meier survival plot based on H3K18la level, and H3K18la was potentially associated with prognosis as determined by a Log-rank test (Fig. 1K).

### Glycolysis inhibition diminishes histone lactylation and inhibits PDAC cell proliferation and migration

**Fig. 2:**
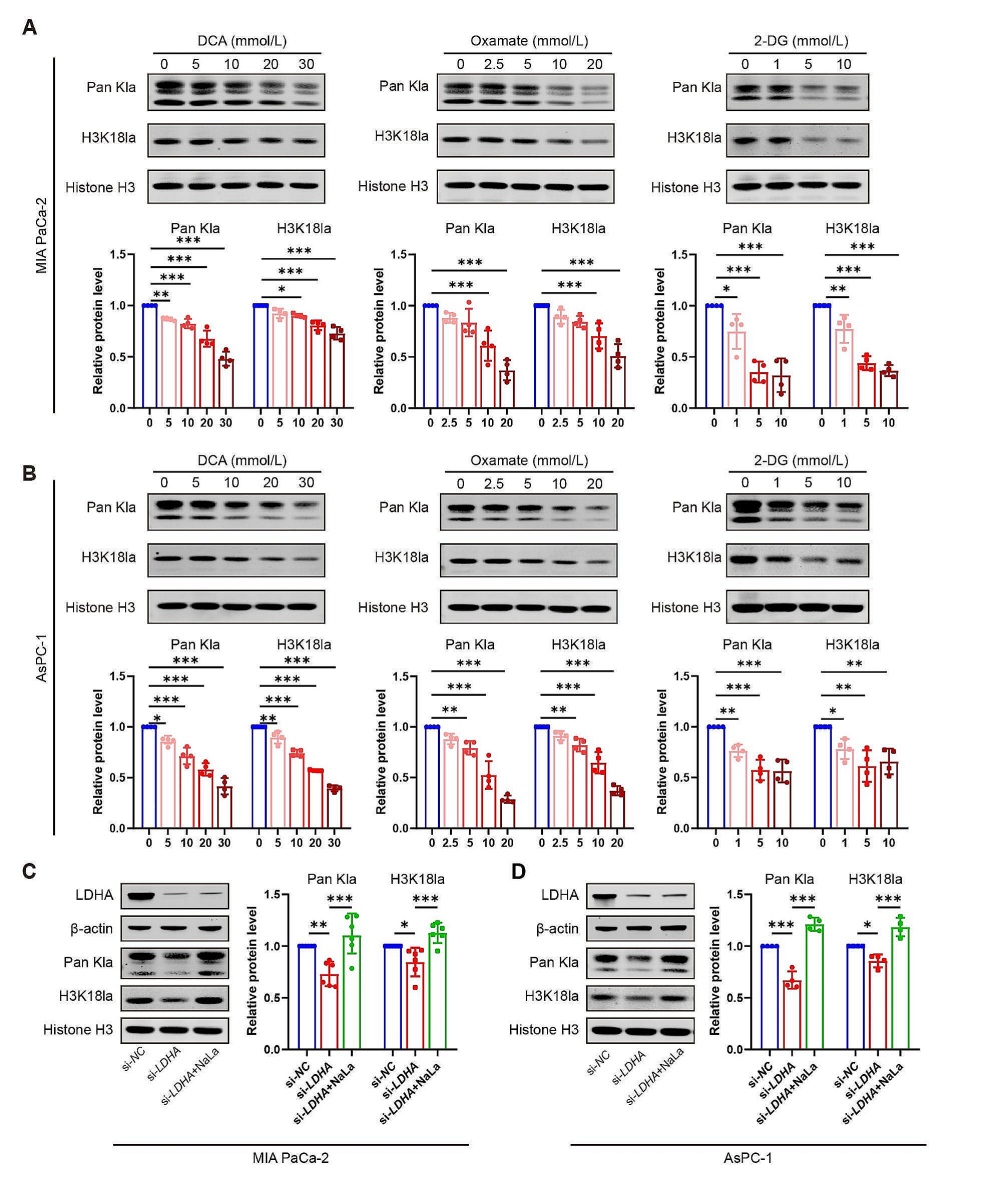
Glycolysis inhibition diminishes histone lactylation. Two PDAC cell lines MIA PaCa-2 and AsPC-1 cells were treated with glycolysis inhibitors DCA (0–30 mmol/L), Oxamate (0–20 mmol/L) or 2-DG (0–10 mmol/L) for 24 h, or transfection with *LDHA* siRNA (si-*LDHA*) or negative control siRNA (si-*NC*) for 48 h with or without sodium lactate (NaLa, 10 mmol/L) treatment. (**A**-**D**) The pan-lysine lactylation (Pan Kla) and H3K18 lactylation (H3K18la) levels were measured by western blot and quantified by using Image J software. *n* = 4 or 6. All data are presented as mean ± SD. Statistical analysis was performed by one-way ANOVA followed by Dunnett’s multiple comparisons test in A-B, or by Tukey’s multiple comparisons test in C-D. ^*^P < 0.05, ^**^P < 0.01, ^***^P < 0.001

To clarify the effects of glycolysis on histone lactylation, we used different kinds of glycolysis inhibitors (DCA, Oxamate and 2-DG) and carried on *LDHA* knockdown in MIA PaCa-2 and AsPC-1 cells respectively. The levels of Pan Kla and H3K18la consistently reduced with increasing dosages of inhibitors (Fig. 2A and B). Furthermore, si-*LDHA* effectively reduced the histone lactylation level, which was significantly increased after Nala treatment (Fig. 2C and D).

**Fig. 3:**
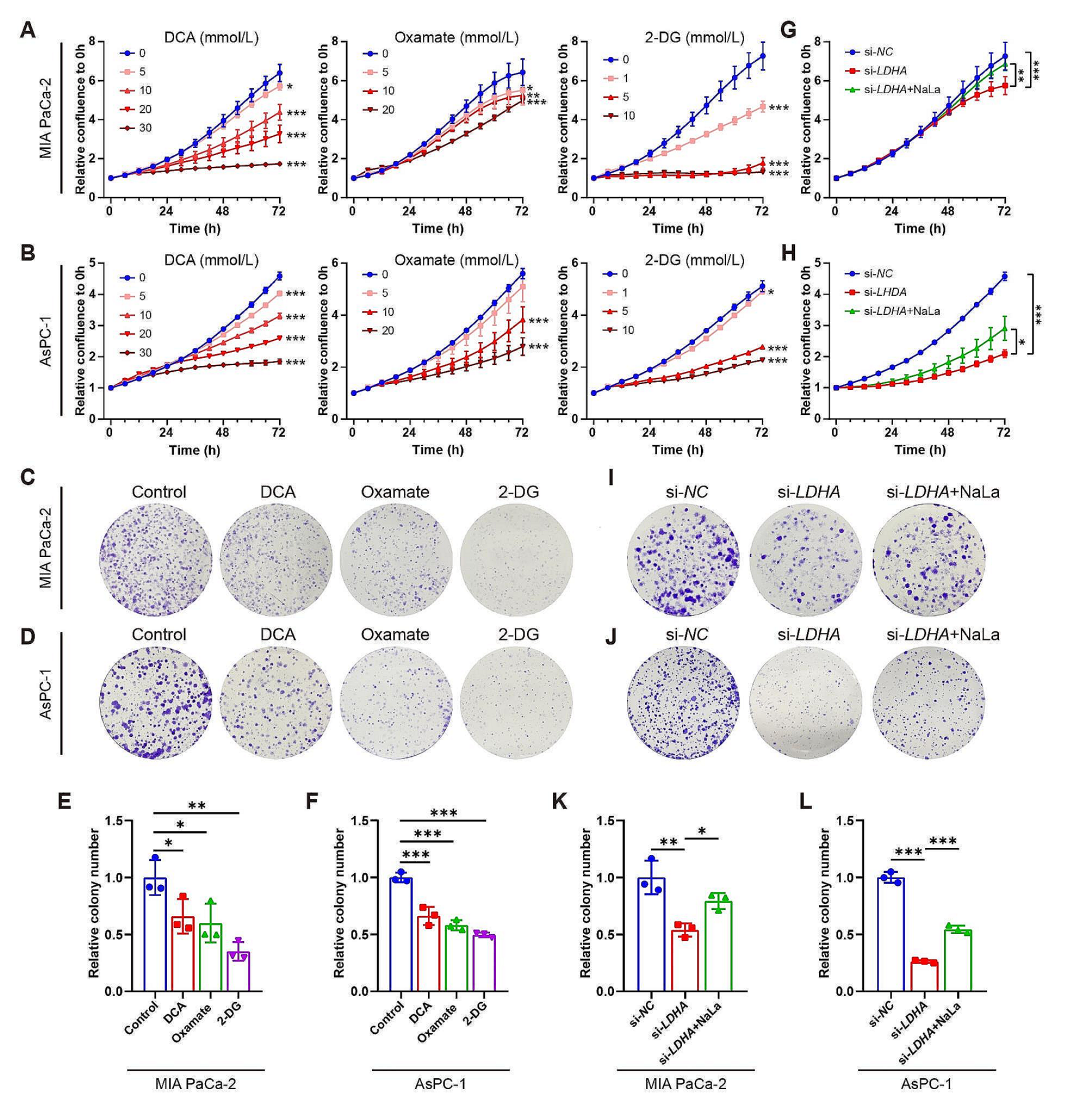
Glycolysis inhibition diminishes histone lactylation and suppresses PDAC cell proliferation. Two PDAC cell lines MIA PaCa-2 and AsPC-1 cells were treated with glycolysis inhibitors DCA (0–30 mmol/L), Oxamate (0–20 mmol/L) or 2-DG (0–10 mmol/L), or transfection with *LDHA* siRNA (si-*LDHA*) or negative control siRNA (si-*NC*) with or without sodium lactate (NaLa, 10 mmol/L) treatment. (**A**-**B**, **G**-**H**) Cell viability was visualized by IncuCyte S3 in MIA PaCa-2 (**A**, **G**) and AsPC-1 (**B**, **H**) cells. *n* = 5, 6 or 3. (**C**-**F**, **I**-**L**) Cell proliferation ability was evaluated by colony formation assays in MIA PaCa-2 (**C**, **E**, **I**, **K**) and AsPC-1 (**D**, **F**, **J**, **L**) cells. *n* = 3. All data are presented as mean ± SD. Statistical analysis was performed by ANOVA followed by Dunnett’s multiple comparisons test in A, B, E, F, or by Tukey’s multiple comparisons test in G, H, K, L. ^*^P < 0.05, ^**^P < 0.01, ^***^P < 0.001

Subsequently, we investigated the biological functions of histone lactylation on cell proliferation. We observed a significant decrease in cell viability and colony formation ability upon treatment with DCA, Oxamate, or 2-DG (Fig. 3A–F). Moreover, *LDHA* silencing exhibited significant inhibition of cell growth and colony formation in both MIA PaCa-2 and AsPC-1 cells, while Nala attenuated the proliferation inhibition effect of *LDHA* silencing (Fig. 3G–L).

**Fig. 4:**
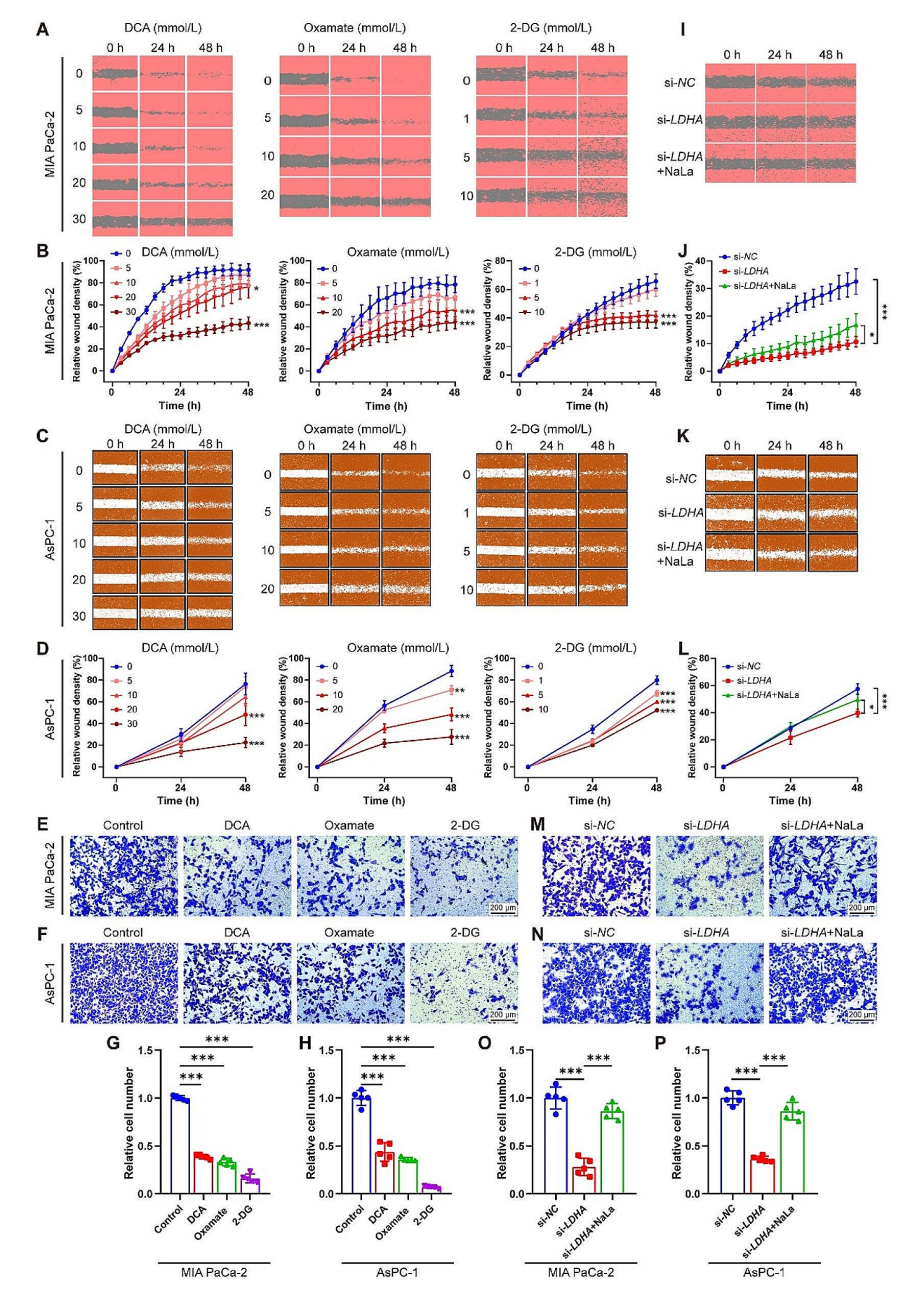
Glycolysis inhibition diminishes histone lactylation and suppresses PDAC cell migration. (**A**-**H**) Two PDAC cell lines MIA PaCa-2 and AsPC-1 cells were treated with glycolysis inhibitors DCA (0–30 mmol/L), Oxamate (0–20 mmol/L) or 2-DG (0–10 mmol/L) for 48 h. The migration ability was evaluated by wound healing and transwell assays. *n* = 4–6. Representative images (**A**, **C**, **E**, **F**) and quantification (**B**, **D**, **G**, **H**). (**I**-**P**) MIA PaCa-2 and AsPC-1 cells were transfected with *LDHA* siRNA (si-*LDHA*) or negative control siRNA (si-*NC*) for 48 h with or without sodium lactate (NaLa,10 mmol/L) treatment. The migration ability was evaluated by wound healing and transwell assays. *n* = 6 or 5. Representative images (**I**, **K**, **M**, **N**) and quantification (**J**, **L**, **O**, **P**). All data are presented as mean ± SD. Statistical analysis was performed by ANOVA followed by Dunnett’s multiple comparisons test in B, D, G, H, or by Tukey’s multiple comparisons test in J, L, O, P. ^*^P < 0.05, ^**^P < 0.01, ^***^P < 0.001

To explore whether lactylation could affect PDAC cell migration, wound healing and transwell assays were performed. Treatments with DCA, Oxamate and 2-DG significantly decreased cell migration in MIA PaCa-2 and AsPC-1 cells, and the higher dose showed more pronounced inhibition effects on the cell migration than the lower dose in the wound healing assay (Fig. 4A–D). Similarly, *LDHA* knockdown inhibited migration capacities compared to the vector group, and Nala addition attenuated the suppressive effect of si-*LDHA* (Fig. 4I–L). Transwell assays also showed that DCA, Oxamate and 2-DG treated MIA PaCa-2 or AsPC-1 cells significantly decreased cell migration (Fig. 4E–H). The same outcomes were observed in *LDHA* knockdown, and Nala counteracted the migratory suppression effect of si-*LDHA* in both cell lines (Fig. 4M–P). Collectively, these findings suggest that histone lactylation plays a crucial role in the initiation and progression of PDAC, while inhibiting histone lactylation may exhibit potential antitumor activity against PDAC.

### Glycolysis inhibition diminishes histone lactylation and suppresses PDAC progression in the PDAC xenograft mouse model

**Fig. 5:**
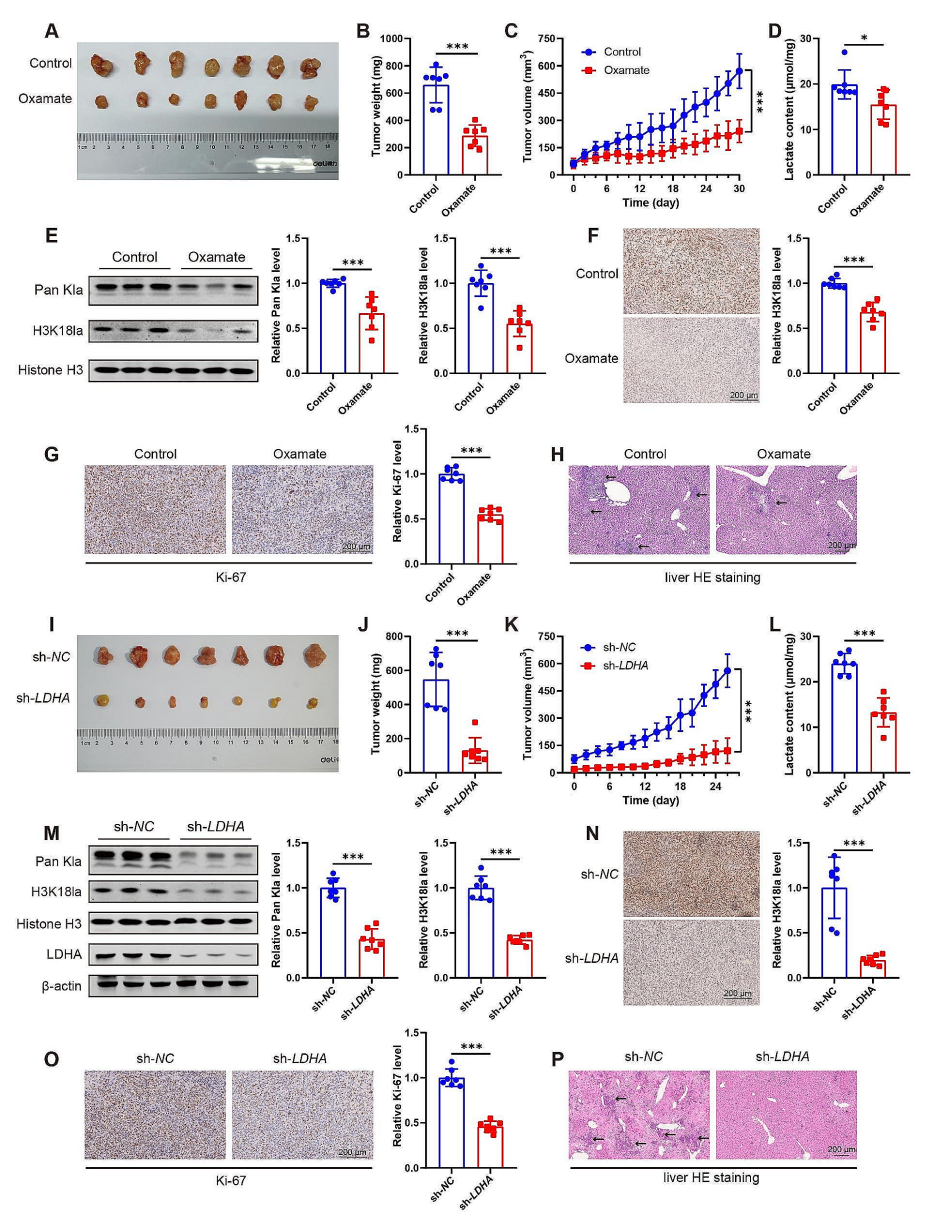
Glycolysis inhibition diminishes histone lactylation and suppresses cancer progression in the transplanted PDAC in nude mice. (**A**-**H**) MIA PaCa-2 cells were injected into nude mice subcutaneously and were intraperitoneally administered Oxamate (750 mg/kg, daily) or vehicle for 30 days. (**I**-**P**) Stable knockdown of *LDHA* (sh-*LDHA*) was mediated by lentivirus in MIA PaCa-2 cells. The sh-*LDHA* cells and the control (sh-*NC*) cells were injected into nude mice subcutaneously. Tumor gross image (**A**, **I**) and tumor weight (**B**, **J**) after sacrifice. Tumor volume assessment during the experiment (**C**, **K**). Lactate content in the tumor tissues (**D**, **L**). The levels of pan-lysine lactylation (Pan Kla) and H3K18 lactylation (H3K18la) were measured by western blot or IHC staining, and quantified by using Image J software (**E**-**F**, **M**-**N**). Cell proliferation was assessed by using IHC staining with Ki-67. Representative images and quantification (**G**, **O**). Tumor metastasis in the liver was evaluated by using HE staining (**H**, **P**). The arrows point to the metastatic tumor lesions. All data are presented as mean ± SD. Statistical analysis was performed by Student’s *t*-test. *n* = 7. ^*^P < 0.05, ^***^P < 0.001

To further evaluate the biological significance of histone lactylation in PDAC progression, a xenograft mouse model was established by implanting MIA PaCa-2 cells subcutaneously into nude mice. Mice were treated with a glycolysis inhibitor Oxamate or vehicle, and the tumor volume was detected every two days. The tumor volume in the Oxamate group was significantly lower than that in the control group during the whole experiment, and the tumor weight also exhibited a dramatic decline in the Oxamate group compared with the control (Fig. 5A–C). To investigate the impact of *LDHA* knockdown on tumor growth, stable sh-*LDHA* and the control MIA PaCa-2 cells were subcutaneously injected into nude mice. Similar to the results of glycolysis inhibitor, tumors in the *LDHA* knockdown group displayed smaller size and lower weight compared to the control groups (Fig. 5I–K).

Next, we detected the effects of glycolysis inhibition on the lactate production and lactylation in the xenograft mouse model. Not surprisingly, the lactate production in the tumor was decreased after Oxamate treatment or *LDHA* knockdown (Fig. 5D and L). Furthermore, diminished levels of Pan Kla and H3K18la were observed along with reduced H3K18la staining intensity in Oxamate-treated tumors (Fig. 5E and F). Similar findings were observed in LDHA inhibition groups: Pan Kla and H3K18la levels were suppressed compared to the control tumor tissues (Fig. 5M and N).

Additionally, a proliferation marker Ki-67 staining and liver HE staining indicated that both Oxamate and sh-*LDHA* treatment decreased the Ki-67 expression and reduced metastatic foci in the liver (Fig. 5G–H, O–P). These results suggested that glycolysis inhibition by the inhibitor or *LDHA* knockdown remarkably decreased tumor proliferation and progression. Altogether, glycolysis inhibition decreases tumor volume and weight, inhibits lactate production and lactylation, and suppresses tumor proliferation and liver metastasis, suggestive of PDAC progression inhibition in the PDAC xenograft mouse model.

### P300 and HDAC2 are potential writer and eraser of histone lactylation in PDAC cells respectively

**Fig. 6:**
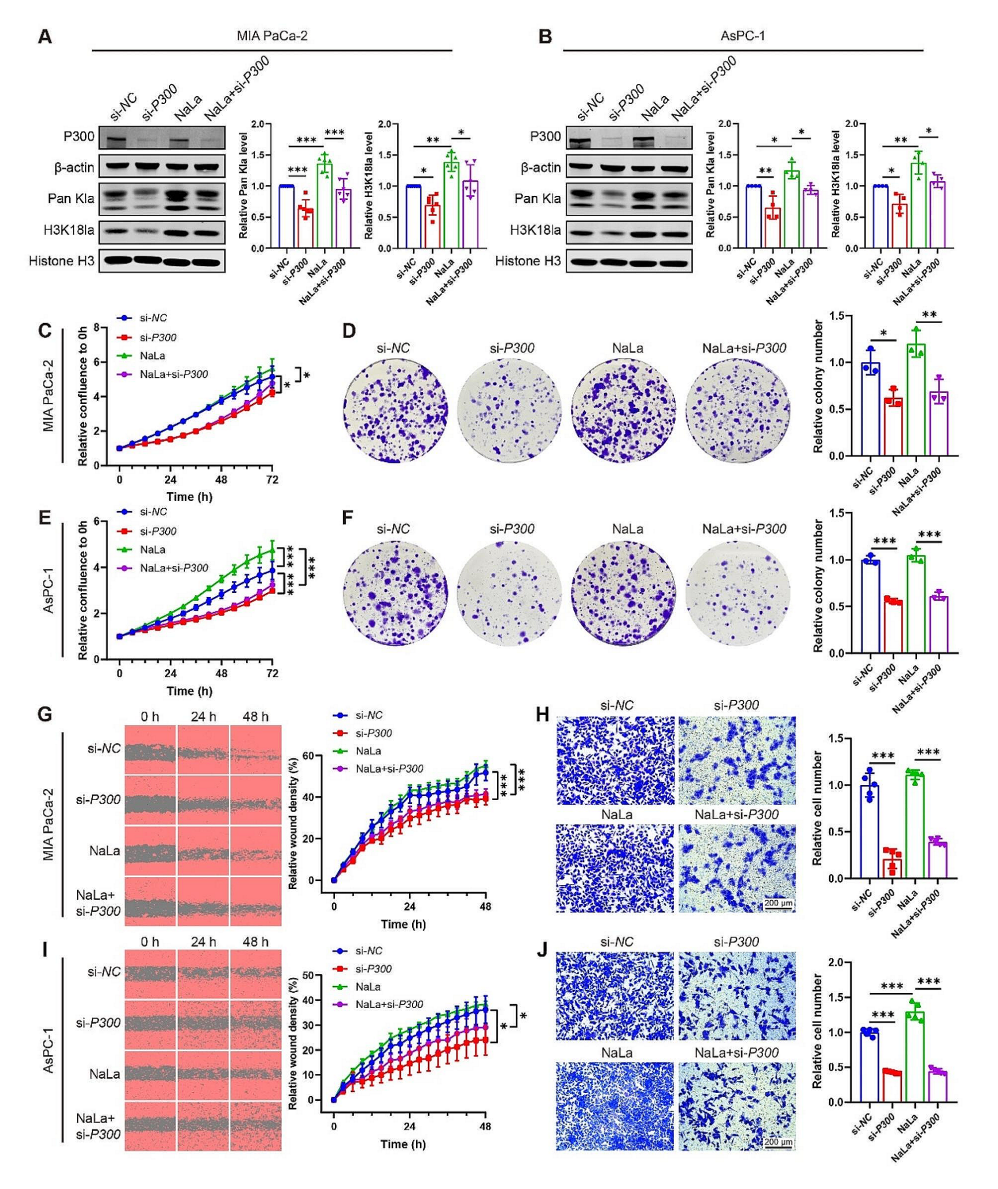
P300 is a potential writer of histone lactylation in PDAC cells. PDAC cell lines MIA PaCa-2 and AsPC-1 cells were transfected with *P300* siRNA (si-*P300* or negative control siRNA (si-*NC*) with or without sodium lactate (NaLa, 10 mmol/L) treatment. (**A**-**B**) The pan-lysine lactylation (Pan Kla) and H3K18 lactylation (H3K18la) levels were measured in MIA PaCa-2 (**A**) and AsPC-1 (**B**) cells. *n* = 6 or 4. Representative images (left panel), and the quantification (right panel). (**C**-**F**) The proliferation ability of MIA PaCa-2 (**C**, **D**) and AsPC-1 (**E**, **F**) cells was detected by using IncuCyte S3 (**C**, **E**) and colony formation assays (**D**, **F**). *n* = 3, 5 or 6. (**G**-**J**) The migration ability of MIA PaCa-2 (**G**, **H**) and AsPC-1 (**I**, **J**) cells were assessed by using wound healing (**G**, **I**) and transwell assays (**H**, **J**). *n* = 4 or 5. All data are presented as mean ± SD. Statistical analysis was performed by ANOVA followed by Tukey’s multiple comparisons test. ^*^P < 0.05, ^**^P < 0.01, ^***^P < 0.001

The acetyltransferase P300 has been reported as a potential writer of histone lactylation [20]. However, its involvement in PDAC progression has been rarely studied in depth. To assess the impact of P300 on lactylation, we used *P300* knockdown (si-*P300*) or C646 (a P300 inhibitor) with or without NaLa treatment in MIA PaCa-2 and AsPC-1 cells. Figure 6A and Fig. S1A demonstrated a significant decrease in both Pan Kla and H3K18la levels in the si-*P300* or C646 group in MIA PaCa-2 cells respectively. Under the condition of NaLa treatment, si-*P300* or C646 still significantly decreased Pan Kla and H3K18la levels (Fig. 6A, S1A). The similar results were also observed in AsPC-1 cells (Fig. 6B, S1B). These results suggest that P300 might be a potential writer of histone lactylation.

Next, we investigated the effects of P300 on cell proliferation and migration. We found that *P300* silencing or C646 treatment in MIA PaCa-2 cells resulted in a substantial reduction in cell proliferation by using IncuCyte S3 and colony formation assays (Fig. 6C and D, S1C-D). In the presence of NaLa, cell viability was also inhibited by *P300* knockdown or C646 (Fig. 6C and D, S1C-D). The efficacy of *P300* knockdown or C646 in suppressing cell proliferation and colony formation was similarly evident in AsPC-1 cells (Fig. 6E and F, S1E-F). Cell migration rate was evaluated by using wound healing and transwell assays. Silencing of *P300* or treatment with C646 significantly impaired migration abilities for both MIA PaCa-2 (Fig. 6G and H, S1G-H) and AsPC-1 cells (Fig. 6I and J, S1I-J). Next, we investigated whether P300 knockdown or C646 treatment could reverse the effect of NaLa on PDAC migration. As expected, cells transfected with si-*P300* or treatment with C646 in combination with NaLa exhibited reduced migration ability compared to those solely treated with NaLa in MIA PaCa-2 (Fig. 6G and H, S1G-H) and AsPC-1 cells (Fig. 6I and J, S1I-J). These results suggest that P300, the potential writer of histone lactylation, promotes progression of PDAC.

To identify the potential eraser of histone lactylation in PDAC, various types of histone deacetylase (HDAC) inhibitors were employed in MIA PaCa-2 and AsPC-1 cells. Intriguingly, treatment with broad-spectrum HDAC inhibitors TSA, rather than sirtuin inhibitor NAM, resulted in an increased global level of histone lactylation. Additionally, the class I HDAC inhibitor CI994 enhanced lactylation, while class IIa HDAC inhibitor TMP195, class IIb HDAC inhibitor Bufexamac, and class IV HDAC inhibitor SIS17 had no such effect (Fig. S2A-B). To further validate the role of each HDAC isozyme, overexpression experiments were conducted in MIA PaCa-2 and AsPC-1 cells. Results demonstrated that overexpression of *HDAC2* significantly decreased the lactylation level, particularly H3K18la, while *HDAC1* and *HDAC3* overexpression did not affect the levels of Pan Kla and H3K18la (Fig. S2C-H). These findings suggest that P300 may act as a potential writer while HDAC2 functions as an eraser for histone lactylation in PDAC.

### H3K18la activates *TTK* and *BUB1B* transcription in PDAC

**Fig. 7:**
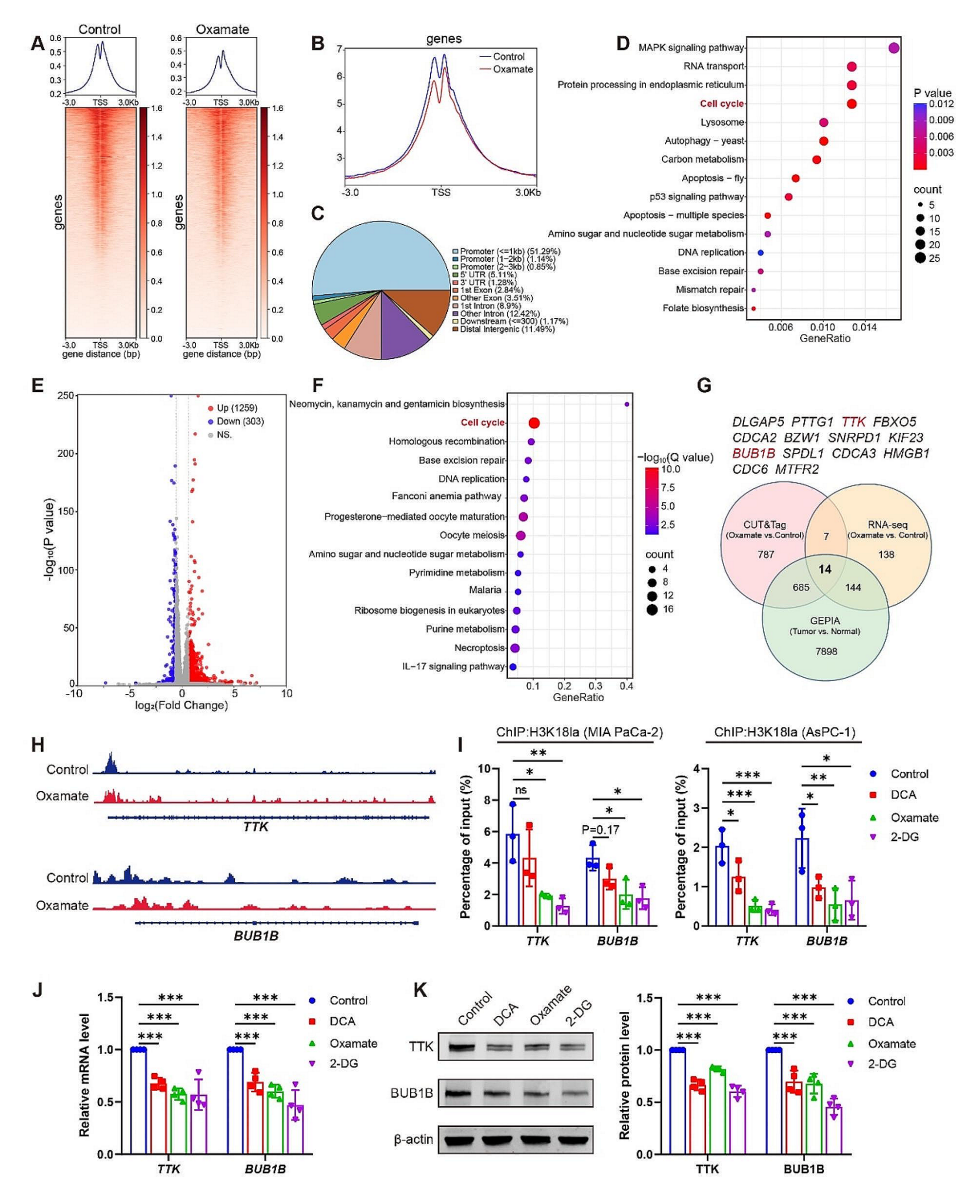
H3K18la activates *TTK* and *BUB1B* transcription in PDAC. (**A**-**H**) MIA PaCa-2 cells were treated with a glycolysis inhibitor Oxamate (10 mmol/L) for 24 h. Cells were collected for CUT&Tag assay to screen the binding sites of H3K18la (**A**-**D, H**) or collected for RNA-seq to screen the downstream genes of lactylation (**E**-**F**). The heatmap showed the distribution of H3K18la peaks in the vicinity of the translation start site (TSS) (**A**-**B**). The distribution of H3K18la on the genome (**C**). KEGG analysis of the promoter region of the H3K18la distribution (**D**). The volcano plot of the differently expressed genes in RNA-seq (**E**). KEGG analysis of the downregulated genes in Oxamate-treated group by RNA-seq (**F**). Combination of CUT&Tag, RNA-seq and GEPIA database to identify the potential downstream targets of H3K18la (**G**). Integrative Genomics Viewer tracks of CUT&Tag showing enriched H3K18la in the promotors of *TTK* and *BUB1B* (**H**). (**I-K**) MIA PaCa-2 or AsPC-1 cells were treated with glycolysis inhibitors DCA (10 mmol/L), Oxamate (10 mmol/L) or 2-DG (5 mmol/L) for 24 h. DNA fragments were immunoprecipitated with the H3K18la antibody and analyzed by qPCR (**I**). *n* = 3. Relative mRNA levels of *TTK* and *BUB1B* in MIA PaCa-2 cells (**J**). *n* = 4. Representative western blot images and quantification of TTK and BUB1B protein levels in MIA PaCa-2 cells (**K**). *n* = 4. All data are presented as mean ± SD. Statistical analysis was performed by one-way ANOVA followed by Dunnett’s multiple comparisons test in I-K. ^*^P < 0.05, ^**^P < 0.01, ^***^P < 0.001; ns, not significant

Next, we tried to determine how H3K18la affect PDAC progression. Histone lactylation, a novel epigenetic modification, has been reported to directly regulate gene transcription from chromatin. Therefore, we performed a CUT&Tag assay using an anti-H3K18la antibody (the original data is in Additional file 3). In the Oxamate-treated group, there was a noticeable reduction in the transcription start site (TSS) region (Fig. 7A and B), with approximately 53% enrichment observed in the promoter regions (Fig. 7C). KEGG analysis of the downregulated peaks in promoter regions specific to Oxamate treatment revealed relevant enrichment in RNA transport pathway, cell cycle, and tumor-related pathways such as MAPK signaling pathway (Fig. 7D).

Additionally, we conducted RNA-seq assay to screen the potential target genes regulated by lactylation (the original data is in Additional file 4). When compared with the control group, Oxamate treatment upregulated 1259 genes and downregulated 303 genes in PDAC cells (Fig. 7E). The KEGG analysis of these downregulated genes also showed enrichment in the cell cycle pathway in Oxamate treatment groups (Fig. 7F). Geno ontology (GO) analysis of both CUT&Tag and RNA-seq analysis also demonstrated enrichment in cell cycle-related processes (Fig. S3A-B).

We searched for genes with high expression in PDAC in GEPIA database and found 8741 genes (log_2_Fold Change > 1, Q value < 0.05). By overlapping the gene sets among CUT&Tag, RNA-seq and GEPIA database, we identified 14 differential expressed genes that exhibited significantly reduced mRNA levels and reduced enrichment of the H3K18la signal at the promoter region upon Oxamate treatment but were upregulated in PDAC tissues (Fig. 7G). Given that both the CUT&Tag and RNA-seq enriched in the cell cycle pathway, we focused on the genes involved in the cell cycle pathway. The results revealed that 4 genes (*CDC6*, *TTK*, *BUB1B*, *PTTG1*) were associated with the cell cycle pathway, predominantly involved in the M phase (*TTK*, *BUB1B*, *PTTG1*) (Fig. S3C). Notably, among them, *TTK* (also known as *MPS1*) and *BUB1B* (also known as *BUBR1*), which are mitotic spindle assembly checkpoint regulators and locate in the upstream of the M phase, are important for accurate chromosome segregation during mitosis [21]. Consequently, we decided to investigate these two genes.

We identified an obvious reduction of peaks at the promoter positions of *TTK* and *BUB1B* in cells treated with Oxamate (Fig. 7H). We used ChIP-qPCR assays to further confirm that H3K18la was enriched at the *TTK* and *BUB1B* promoters, which were reduced by glycolysis inhibitors DCA, Oxamate, or 2-DG (Fig. 7I). Besides, the mRNA and protein levels of TTK and BUB1B were significantly downregulated by different inhibitors of glycolysis in MIA PaCa-2 cells (Fig. 7J and K). These results suggest that the activation of *TTK* and *BUB1B* might be involved in the H3K18la-mediated PDAC promotion.

### The high levels of TTK and BUB1B are associated with the malignancy of PDAC

**Fig. 8:**
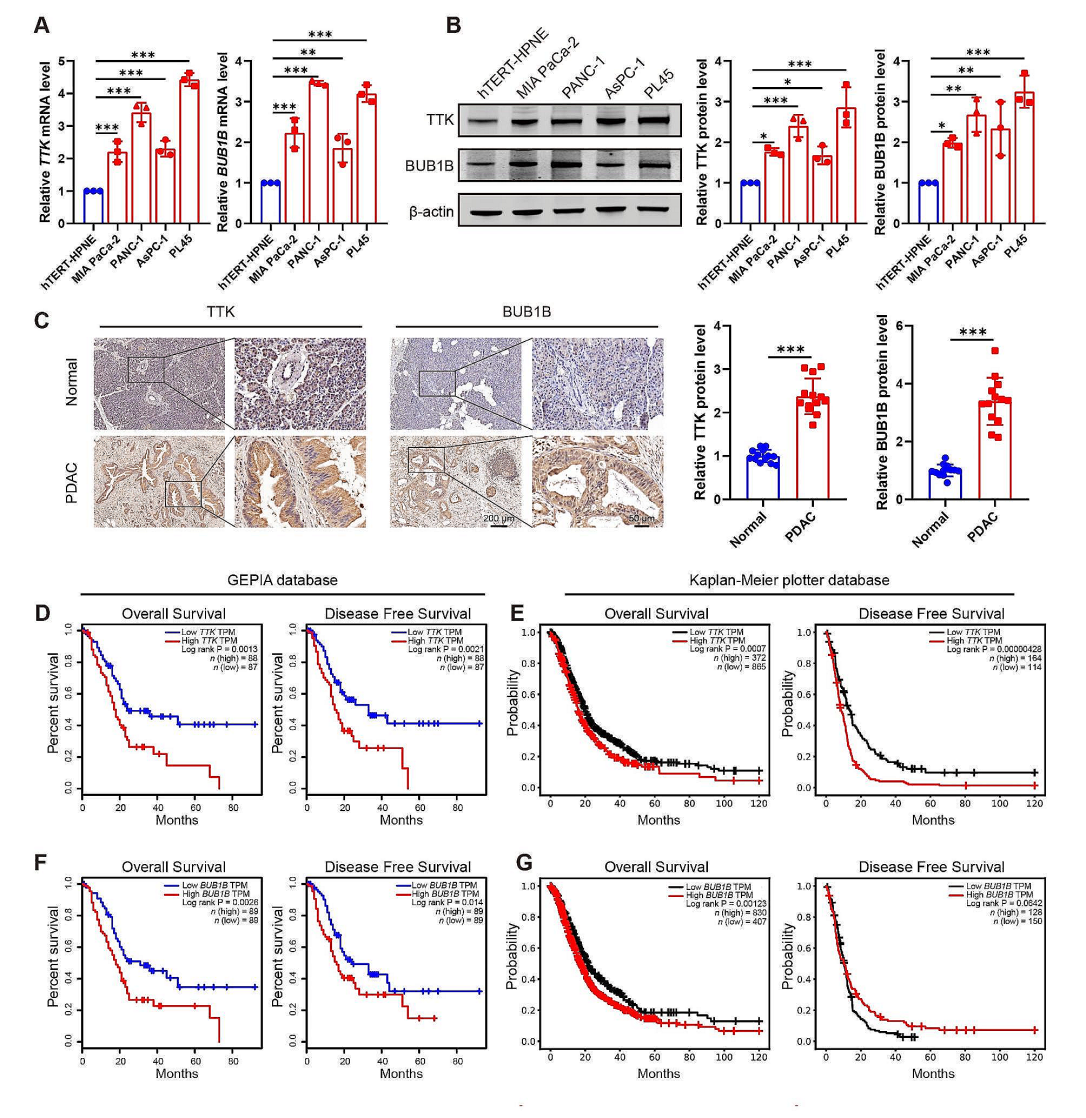
The high levels of TTK and BUB1B are associated with the malignancy of PDAC. (**A**) Relative mRNA levels of *TTK* and *BUB1B* were detected in human pancreatic ductal epithelial cell line hTERT-HPNE and four different PDAC cell lines (MIA PaCa-2, PANC-1, AsPC-1, and PL45 cells) by RT-qPCR. *n* = 3. (**B**) Representative western blot images and quantification of TTK and BUB1B protein levels in the above cell lines. *n* = 3. (**C**) TTK and BUB1B expression levels were visualized by IHC staining in PDAC and para-carcinoma tissues on the left panel. Statistical results were shown on the right panel. *n* = 13. (**D**-**G**) Kaplan-Meier curves of overall survival and disease-free survival in PDAC patients with low and high *TTK* or *BUB1B* expression in the GEPIA database (**D**, **F**) and Kaplan-Meier plotter database (**E**, **G**). All data are presented as mean ± SD. Statistical analysis was performed by one-way ANOVA followed by Dunnett’s multiple comparisons test in A, B, or by Student’s *t*-test in C, or by Log-rank test in D-G. ^*^P < 0.05, ^**^P < 0.01, ^***^P < 0.001. TPM, transcripts per million

To investigate the effects of TTK and BUB1B in PDAC progression, we first detected their expression in PDAC. The transcription and protein levels of TTK and BUB1B were significantly higher in four PDAC cell lines (MIA PaCa-2, PANC-1, AsPC-1, and PL45 cells) in comparison with hTERT-HPNE cells (Fig. 8A and B). IHC analysis revealed a significant increase in TTK and BUB1B expression in PDAC tissues compared to para-carcinoma tissues (Fig. 8C). GEPIA and GEO database also confirmed that TTK and BUB1B expression were elevated in PDAC tissues compared with normal tissues (Fig. S4A-D). Additionally, analysis with GEPIA and Kaplan-Meier Plotter database demonstrated that PDAC patients with high TTK or BUB1B expression had shorter overall survival time or disease-free survival time than those with lower expression levels (Fig. 8D-G).

### *TTK* and *BUB1B* deletion suppresses PDAC malignancy while *TTK* overexpression partially prevents the anticancer effects of lactylation inhibition

**Fig. 9:**
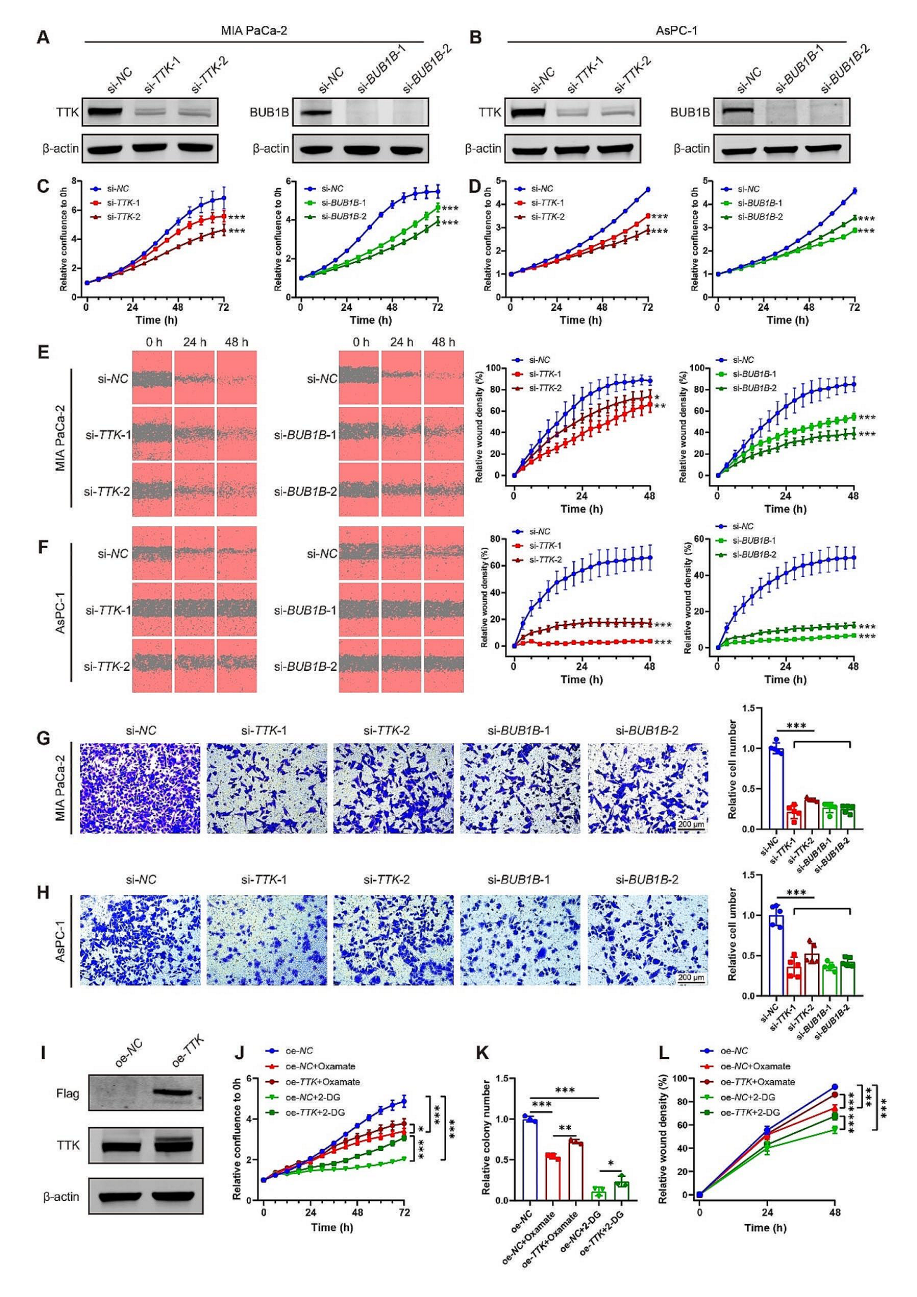
*TTK*/*BUB1B* knockdown suppresses PDAC malignancy, and *TTK* overexpression partially attenuates the anticancer effects of lactylation inhibition. (**A**-**H**) MIA PaCa-2 and AsPC-1 cells were transfected with *TTK* siRNA (si-*TTK*), *BUB1B* siRNA (si-*BUB1B*) or negative control siRNA (si-*NC*). The efficiency of *TTK* and *BUB1B* silencing was detected by western blot (**A**-**B**). The proliferation was assessed using IncuCyte S3 (**C**-**D**). *n* = 6. The migration ability was assayed by wound healing assays and transwell assays (**E**-**H**). *n* = 4–6. (**I**-**L**) MIA PaCa-2 cells were transfected with *TTK* overexpression plasmid (oe-*TTK*), or negative control plasmid (oe-*NC*), and then treated with glycolysis inhibitors Oxamate (10 mmol/L) or 2-DG (2 mmol/L). The efficiency of TTK overexpression was determined by western blot (**I**). The cell proliferation was assessed using IncuCyte S3 (**J**) and colony formation assays (**K**). *n *= 6 or 3. The migration ability was assessed by wound healing assays (**L**). *n* = 5. All data are presented as mean ± SD. Statistical analysis was performed by ANOVA followed by Dunnett’s multiple comparisons test in C-H, or by Tukey’s multiple comparisons test in J-L. ^*^P < 0.05, ^**^P < 0.01, ^***^P < 0.001

To assess the function of TTK and BUB1B in PDAC, we used siRNA transfection to knockdown *TTK* and *BUB1B* expression in MIA PaCa-2 and AsPC-1 cells (Fig. 9A and B, S5A-D). Knockdown of either *TTK* or *BUB1B* resulted in a significant reduction in cell viability for both MIA PaCa-2 and AsPC-1 cells compared to the control group (Fig. 9C and D). Wound healing assays (Fig. 9E and F) as well as transwell assays (Fig. 9G and H) indicated that silencing *TTK* or *BUB1B* suppressed cell migration.

To investigate the effect of TTK in H3K18la-mediated PDAC progression, MIA PaCa-2 cells were overexpressed with TTK (Fig. 9I) and treated with glycolysis inhibitors. Compared with negative control, *TTK* overexpression didn’t promote the ability of proliferation or migration of MIA PaCa-2 cells (Fig. S5E-F). This might be due to the fact that the level of TTK was already extremely high in PDAC [22, 23], and its ability to promote tumor progression was saturated. Similar to the results above mentioned, glycolysis inhibitors Oxamate and 2-DG inhibited cell growth and migration in MIA PaCa-2 cells. Interestingly, *TTK* overexpression together with glycolysis inhibitors compromised the anticancer effects of glycolysis inhibitors on cell growth inhibition and migration suppression (Fig. 9J-L). These results suggest that TTK and BUB1B are involved in the PDAC progression, and TTK participates in the H3K18la-mediated PDAC malignancy.

### A positive feedback loop between H3K18la target genes and glycolysis

**Fig. 10:**
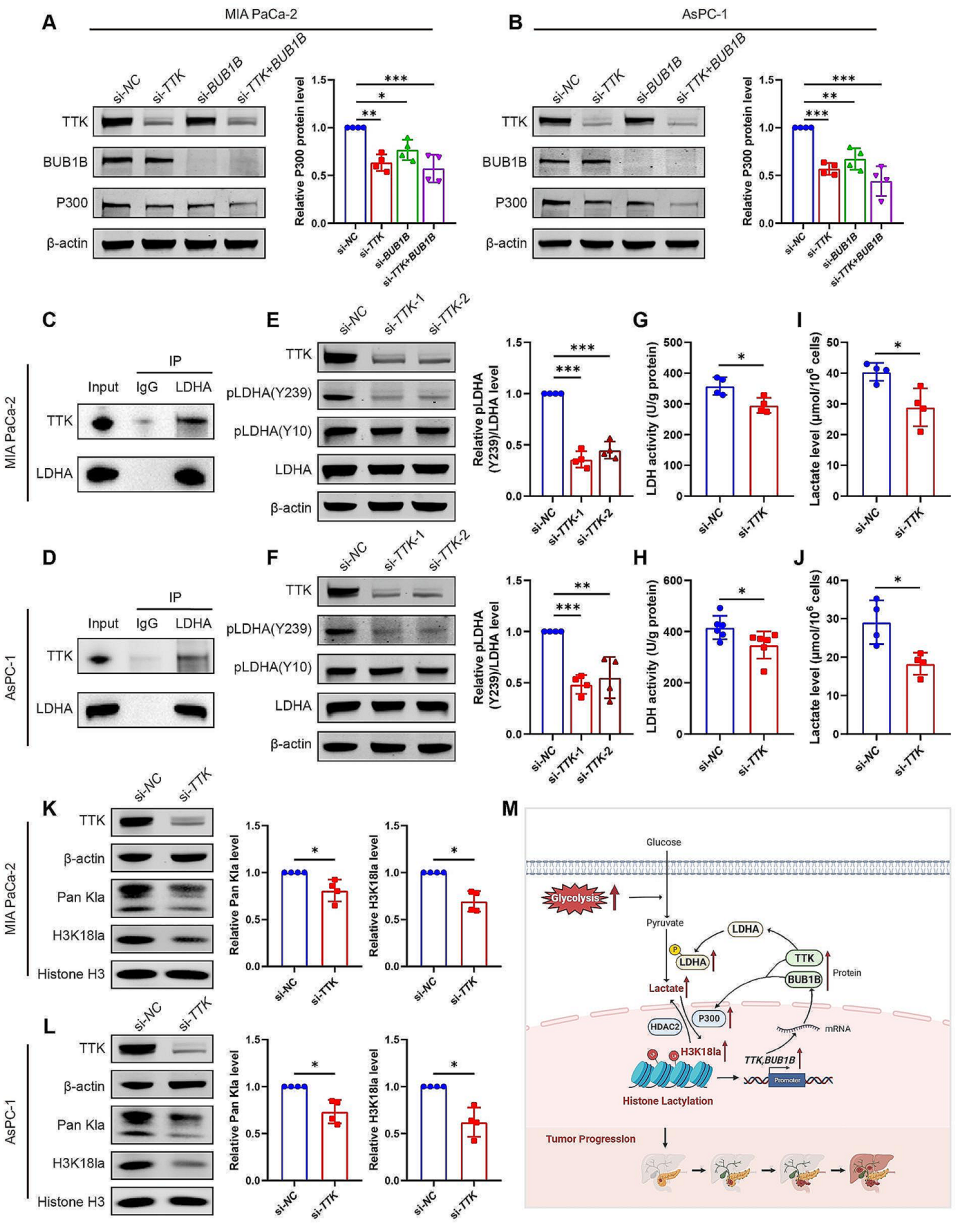
A positive feedback loop between H3K18la target genes (*TTK* and *BUB1B*) and glycolysis. (**A**-**B**) MIA PaCa-2 (**A**) and AsPC-1 (**B**) cells were transfected with negative control siRNA (si-*NC*), *TTK* siRNA (si-*TTK*) and/or *BUB1B* siRNA (si-*BUB1B*). The protein levels were detected by western blot and quantified. *n* = 4. (**C**-**D**) The interactions between TTK and LDHA in MIA PaCa-2 (**C**) and AsPC-1 (**D**) cells were confirmed by co-immunoprecipitation (Co-IP). (**E**-**L**) MIA PaCa-2 and AsPC-1 cells were transfected with si-*TTK* or si-*NC*. (**E**-**F**) The levels of phosphorylated LDHA at Y239 and Y10 in MIA PaCa-2 (**E**) and AsPC-1 (**F**) cells were detected by western blot and quantified. *n* = 4. (**G**-**H**) LDHA activity assays in MIA PaCa-2 (**G**) and AsPC-1 (**H**) cells. *n* = 4 or 6. (**I**-**J**) The lactate level in cell culture supernatants. *n* = 4. (**K**-**L**) The pan-lysine lactylation (Pan Kla) and H3K18 lactylation (H3K18la) levels were measured by western blot and quantified using Image J software. *n* = 4. All data are presented as mean ± SD. Statistical analysis was performed by one-way ANOVA followed by Tukey’s multiple comparisons test in A-B, or by Dunnett’s multiple comparisons test in E-F, or by Student’s *t*-test in G-L. ^*^P < 0.05, ^**^P < 0.01, ^***^P < 0.001. (**M**) The main findings of this article. The glycolysis-H3K18la-TTK/BUB1B positive feedback loop exacerbates dysfunction in PDAC. This image was created with the help of BioRender (https://www.biorender.com)

It has been reported that the expression of BUB1B was positively related to the cell cycle and glycolysis in lung adenocarcinoma. *BUB1B* knockdown could decrease the glycolysis-related genes, including solute carrier family 2 number 1, *LDHA*, pyruvate kinase M 2, and hexokinase 2, suggesting that BUB1B acts as an activator in glycolytic metabolism to promote lung adenocarcinoma [24]. Therefore, it was reasonable to hypothesize that BUB1B might serve as a crucial regulator of glycolysis, supporting uncontrolled proliferation in PDAC. Interestingly, to our attention, the protein levels of P300 were significantly reduced in PDAC cells transfected with si-*TTK*, si-*BUB1B*, or a combination of both (Fig. 10A and B). Considering P300 was proved to be a writer of histone lactylation in the beforementioned results, we concluded that during PDAC progression, not only the glycolysis-histone lactylation-TTK/BUB1B pathway existed, but also TTK/BUB1B played positive feedback in histone lactylation, which further exacerbate the malignancy.

Furthermore, we tried to determine whether TTK/BUB1B could affect glycolysis in PDAC. LDHA plays a pivotal role in the synthesis of lactate, and it drives cancer cells’ preference for aerobic glycolysis in PDAC [25]. The potential phosphorylation sites of LDHA included tyrosine 10 (Y10) and tyrosine 239 (Y239) as predicted by PhosphoSitePlus (https://www.phosphosite.org/homeAction, Fig. S6A). Considering TTK’s dual specificity kinase activity towards both tyrosine and serine/threonine residues [26], we were interested in determining whether TTK has the capability to phosphorylate LDHA. Thus, we investigated whether there is an in vivo interaction between TTK and LDHA by using Co-IP assays. IP with LDHA antibody followed by western blot with TTK antibody confirmed the interaction between TTK and LDHA in MIA PaCa-2 and AsPC-1 cells (Fig. 10C and D). Furthermore, the knockdown of *TTK* significantly suppressed the expression of phosphorylated LDHA at Y239 (Fig. 10E and F). There was only a slight reduction for phosphorylation at Y10 after *TTK *knockdown, but the results had no statistical significance (Fig. 10E and F, S6B-C). We also detected the activity of LDH, and found inhibition of TTK could suppress the LDH activity (Fig. 10G and H), These results suggest that TTK interacts with LDHA, and affects its phosphorylation and activity. Next, we evaluated the downstream of LDHA phosphorylation to investigate the effects of TTK on glycolysis in PDAC. Results showed *TTK* knockdown reduced the production of lactate (Fig. 10I and J), and decreased the levels of Pan kla and H3K18la (Fig. 10K and L) in both MIA PaCa-2 and AsPC-1 cells. These results suggest that TTK plays a role in positive feedback between glycolysis and lactylation. Altogether, we find a positive feedback loop between H3K18la target genes TTK/BUB1B and glycolysis/lactylation.

## Discussion

Our investigation into PDAC revealed a notable increase in lactate accumulation within the tumor microenvironment, which subsequently served as substrates for histone lactylation, notably H3K18la, which emerged as a key driver of PDAC tumorigenesis and progression and was correlated significantly with adverse clinical outcomes. P300 was identified as a writer and HDAC2 as an eraser of histone lactylation in PDAC cells. Furthermore, H3K18la stimulated *TTK* and *BUB1B* transcription, thereby promoting cell cycle progression and tumorigenesis in PDAC. Intriguingly, knockdown of *TTK* and *BUB1B* resulted in reduced P300 expression, and *TTK* knockdown inhibited LDHA phosphorylation at Y239, leading to decreased lactate production and histone lactylation level, establishing a feedback loop among these molecules. This glycolysis-H3K18la-TTK/BUB1B positive feedback loop presents a promising avenue for potential supplementary therapeutic approaches in PDAC treatment. In summary, our study provides critical insights into the role of histone lactylation in PDAC pathogenesis, emphasizing potential therapeutic targets for this challenging disease.

PDAC is one of the most lethal types of cancer and a significant challenge in the field of cancer medicine. Metabolic disorders are risk factors for PDAC progression. Reprogramming cellular metabolism to support proliferation and various cellular processes is considered a key hallmark of cancer [27]. Glycolysis plays a central role in the metabolic reprogramming of PDAC cells and supports malignant behaviors. Aerobic glycolysis, commonly recognized as the Warburg effect, is characterized by the preferential generation of energy through glycolysis instead of oxidative phosphorylation, even in the presence of adequate oxygen levels, which results in substantial production of lactate [28]. This phenomenon emerges as a hallmark of tumor cells, particularly in PDAC. Therefore, targeting the Warburg effect holds paramount importance in exploring novel therapeutic strategies against PDAC [29]. Lactate was previously regarded solely as a byproduct of glycolytic metabolism until Faubert et al. discovered that it could be reused as the primary carbon source for the mitochondrial tricarboxylic acid cycle [30]. Zhang et al., on their part, provided evidence that lactate has the potential to act as an epigenetic regulator by modifying histone lysine residues through lactylation [13]. In recent years, studies have revealed the participation of lactylation in processes such as tumorigenesis [19, 20], heart failure [31], sepsis [32] and other diseases. However, studies on histone lactylation modification in PDAC have not been reported yet till now. In our present study, we observed lactate accumulation in the tumor microenvironment of PDAC, which served as substrates for histone lactylation. We have revealed that histone lactylation, particularly H3K18la, could drive tumorigenesis and progression in PDAC in vitro and in vivo for the first time. Furthermore, the high level of lactylation showed a strong correlation with poor clinical outcomes. All these results suggest that lactylation participates in the development of PDAC. However, we could not prove the direct connection between H3K18la and PDAC development, owing that the complex and dynamic nature of histone modifications makes them less likely to be affected by point mutations in a single base. This is a common limitation in studies aimed at demonstrating the direct association of histone modifications (including lactylation, acetylation and phosphorylation) with diseases [19, 20, 33–35]. Besides, the effects of lactylation in other histone or non-histone proteins are also interesting.

‘Writer’ and ‘eraser’ play crucial regulatory roles in epigenetic modifications. A writer is an enzyme responsible for adding or introducing a specific modification to a substrate molecule, while an eraser is an enzyme responsible for removing or reversing a specific post-translational modification from a substrate molecule. Currently, it has been noted that acetyltransferase P300 [20] and GCN5 [33] could act as histone lactylation writers. while deacetylase HDAC1-3 [36] along with SIRT3 [37] serve as lactylation erasers. Here, by using inhibitors and function experiments, we identified P300 and HDAC2 as the potential writer and eraser respectively involved in regulating histone lactylation in PDAC cells. By silencing or inhibiting P300 to disrupt histone lactylation, we were able to inhibit cell proliferation and migration ability in PDAC cells in a high lactate environment. Notably, lactate intervention promoted proliferation and migration abilities in AsPC-1 cells, but not in MIA PaCa-2 cells. This discrepancy may arise from the higher lactate production in MIA PaCa-2 cells compared to AsPC-1 cells [38], which is sufficient to promote proliferation and migration. Another question that needs to be noted is that when compared with *P300* knockdown alone, NaLa supplementation together with *P300* knockdown seemed to increase histone lactylation. This is because we only did the knockdown rather than the knockout. Upon the extremely high level of the upstream NaLa, even the writer (P300) was largely limited, the downstream (histone lactylation) might also be upregulated. Also, P300 might not be the sole writer of histone lactylation, and there might be other writers participating in the process. Besides, since P300 also acts as a writer protein for diverse histone acylations including acetylation, crotonylation, and β-hydroxybutyrylation [39, 40], we can’t rule out whether P300-guided other acylation modifications participate in the malignant phenotypes, and further investigations are still needed.

It has been reported that Class I HDAC, especially HDAC1 and HDAC3, are the primary erasers of histone lactylation in cells [41]. In hepatocellular carcinoma, the majority of lactylation sites exhibit positive correlations with HDAC1-3, and knockdown of *HDAC3* in HepG2 cells leads to an increase in lactylation intensity [42]. In addition, the sirtuin family has also been reported to participate in the lactylation. Under hypoxic conditions, mitochondrial alanyl-tRNA synthetase accumulates and lactylates mitochondrial proteins, thereby inhibiting oxidative phosphorylation. However, this process can be reversed by SIRT3, which suggests that SIRT3 may act as an eraser of mitochondrial protein lactylation [37]. Here, our intervention with deacetylase inhibitors demonstrated the potential role of Class I HDAC function as delactylase and overexpression experiments indicated that HDAC2 might function as an eraser in PDAC cells. However, understanding the specific mechanism of HDAC2 in PDAC development, as well as elucidating the roles of other HDAC families, requires further investigation in the future.

Increased histone lactylation in the promoter regions has been verified to induce the expression of target genes, including Arginase 1 (*Arg1*), N6-methyladenosinereader protein *YTHDF2* in different cell types [13, 20]. In the present study, we revealed H3K18la activated *TTK* and *BUB1B* transcription in PDAC cells to drive the cell cycle and accelerate tumorigenesis for the first time. The cell cycle pathway is an evolutionarily conserved process necessary for mammalian cell growth. Tumor cells accumulate cell cycle aberrations that result in unscheduled proliferation, genomic instability. Numerous therapeutic strategies have been proposed for targeting the cell cycle in cancer. In this study, we showed that mitotic spindle assembly checkpoint regulators TTK and BUB1B were highly expressed in human pancreatic cancer tissues and worked as prognostic factors in PDAC, which are in consistent with previous reports [22, 23, 43]. Although the mechanisms of several checkpoints have been well established, however, the functional significance of TTK and BUB1B in PDAC cells has not been analyzed in depth. By using knockdown and overexpression together with glycolysis inhibitors, we confirmed the effects of TTK and BUB1B in the H3K18la-mediated PDAC malignancy. We provided a novelty explanation for targeting critical regulators of genomic stability and the mitotic checkpoint TTK and BUB1B in PDAC.

More and more researchers have confirmed that metabolic rewiring and epigenetic remodeling are closely linked and reciprocally regulate each other [44]. In clear cell renal cell carcinoma, inactive von Hippel-Lindau leads to H3K18la, which activates the promoter of platelet-derived growth factor receptor β; and the latter signaling, in turn, stimulates H3K18la, establishing an oncogenic positive feedback loop [45]. Microglial cells exhibit significant enrichment of histone H4 lysine 12 lactylation in brain specimens from patients with Alzheimer’s disease, which target the promoter regions of genes associated with glycolysis, thereby inducing their transcription and promoting lactate production. This establishes a positive feedback loop involving metabolism-epigenetics-metabolism, which exacerbates microglial metabolic dysregulation and functional impairments [46]. However, a comprehensive understanding of metabolic reprogramming and histone lactylation in PDAC is still lacking. In this study, we found the knockdown of *TTK* and *BUB1B*, the target genes of H3K18la, could reduce the expression of P300 in different PDAC cell lines. As a writer of histone lactylation, P300 could further inhibit histone lactylation, which formed a feedback loop. Our study delivered a new important supplement about positive feedback regulation between TTK, BUB1B and P300. Besides, LDHA regulates the last step of glycolysis which generates lactate, which is mainly expressed in tissues with high glycolysis, such as tumors [47]. The high lactate level and glycolysis induced histone lactylation, which could induce transcriptional activation in different PDAC cells. Interestingly, knockdown of *TTK*, a target gene for H3K18la, could feedback inhibit the phosphorylation of LDHA at Y239, which further suppressed the activity of LDH and the lactate production, thus decreasing the levels of Pan Kla and H3K18la. The phosphorylation of LDHA has been reported to influence LDHA activity, which is mainly modulated by Y10 [48–50]. However, in our system, we observed for the first time that *TTK* knockdown inhibited the activation of LDHA through Y239 phosphorylation. Altogether, our study establishes a positive feedback loop of glycolysis, histone lactylation and the genes of cell cycle, which adds a new mechanism of PDAC and provides a clue for the treatment of PDAC.

## Conclusion

In summary, our study delivers a new exploration and important supplement of metabolic reprogramming-epigenetic regulation. It can be concluded that lactate promotes cell proliferation and migration at least in part through histone lactylation, especially H3K18la, and this process is mediated by TTK and BUB1B, which in turn enhance the glycolysis, and increase lactylation. Further investigation is needed to elucidate H3K18la-TTK/BUB1B epigenetic reprogramming-linked glycolysis related to PDAC progression. These results may lead to the design of potential therapeutic strategies that are both necessary and pressing for the treatment of PDAC.

### Electronic supplementary material

Below is the link to the electronic supplementary material.

Supplementary Material 1

Supplementary Material 2

Supplementary Material 3

Supplementary Material 4

## Data Availability

The analyzed data sets generated during the study are available from the corresponding authors on reasonable request.
