# Supplementary material for: Positive feedback regulation between glycolysis and histone lactylation drives oncogenesis in pancreatic ductal adenocarcinoma
Source: Mol Cancer. 2024 May 6;23:90. doi: 10.1186/s12943-024-02008-9 (PMC11071201; doi:10.1186/s12943-024-02008-9)
Supplement: Supplementary file 1 — Supplementary Material 1 [file 12943_2024_2008_MOESM1_ESM.docx]

**Supplementary materials**

**Supplementary tables**

**Table S1. The sequences of siRNA and shRNA used in this study**

| **siRNA or shRNA** | **Sequence (5'-3')** |
| --- | --- |
| si-negative control | UUCUCCGAACGUGUCACGUTT |
| si-*TTK*-1 | CCAGAGGACAGACUACUAATT |
| si-*TTK*-2 | GGAUCUAAACCAAGUGGAATT |
| si-*BUB1B*-1 | GCAGCAGAAACGGGCAUUUTT |
| si-*BUB1B*-2 | GCAAUGAGCCUUUGGAUAUTT |
| si*-LDHA* | GCUACACAUCCUGGGCUAUTT |
| si*-P300* | CCGGUGAACUCUCCUAUAATT |
| sh-negative control | TTCTCCGAACGTGTCACGT |
| sh-*LDHA* | GCTACACATCCTGGGCTAT |

**Table S2. Antibodies used in this study**

| **Antibody** | **Source** | **Identifier** | **Dilution (Application)** |
| --- | --- | --- | --- |
| Anti-L-Lactyl Lysine Rabbit mAb | PTM Bio Inc | Cat#PTM-1401RM  RRID: AB_2942013 | 1:1000 (WB) |
| Anti-Lactyl-Histone H3 (Lys18) Rabbit mAb | PTM Bio Inc | Cat#PTM-1406RM  RRID: AB_2909438 | 1:2000 (WB)  1:500 (IHC) |
| Anti-Lactyl-Histone H3 (Lys18) Rabbit mAb | PTM Bio Inc | Cat#PTM-1427RM  RRID: AB_3076698 | 1:50 (CUT&Tag) |
| Anti-Histone H3  Rabbit pAb | Abcam | Cat#ab1791  RRID: AB_302613 | 1:5000 (WB) |
| Anti-KAT3B/P300  Rabbit mAb | Abcam | Cat#ab275378  RRID: AB_2935873 | 1:1000 (WB) |
| Anti-DDDDK Tag (Binds to FLAG tag sequence)  Rabbit mAb | Abcam | Cat#ab205606  RRID: AB_2916341 | 1:10000 (WB) |
| Anti-LDHA Rabbit mAb | Cell Signaling Technology | Cat#3582  RRID: AB_2066887 | 1:1000 (WB) |
| Anti-LDHA Rabbit pAb | Proteintech | Cat#19987-1-AP  RRID: AB_10646429 | 1:150 (IP) |
| Anti-HDAC1 Mouse mAb | Proteintech | Cat#66085-1-Ig  RRID: AB_11232033 | 1:10000 (WB) |
| Anti-HDAC2 Mouse mAb | Proteintech | Cat#67165-1-Ig  RRID: AB_2882461 | 1:20000 (WB) |
| Anti-HDAC3 Rabbit pAb | Proteintech | Cat#10255-1-AP  RRID: AB_2279733 | 1:1000 (WB) |
| Anti-TTK Rabbit pAb | Proteintech | Cat#10381-1-AP  RRID: AB_2211979 | 1:1000 (WB)  1:500 (IHC) |
| Anti-BUB1B Rabbit pAb | Proteintech | Cat#11504-2-AP  RRID: AB_2066073 | 1:1000 (WB)  1:200 (IHC) |
| Anti-Phospho-LDHA (Tyr10) Rabbit pAb | Thermo Fisher Scientific | Cat#PA5-117223  RRID: AB_2901853 | 1:1000 (WB) |
| Anti-Phospho-LDHA (Tyr239) Rabbit pAb | Thermo Fisher Scientific | Cat#PA5-105446  RRID: AB_2816874 | 1:1000 (WB) |
| Anti-β-actin Mouse mAb | Applygen | Cat#C1313  RRID: AB_3076668 | 1:10000 (WB) |
| Anti-Ki67 Moues mAb | Servicebio | Cat#GB121141  RRID: AB_3083641 | 1:600 (IHC) |
| Anti-IgG Rouse mAb | Biodragon | Cat#BF01006 | 1:150 (IP) |
| IRDye 800CW Goat Anti-Rabbit IgG Secondary Antibody | LI-COR Biosciences | Cat#926-32211  RRID: AB_621843 | 1:10000 (WB) |
| IRDye 800CW Goat Anti-Mouse IgG Secondary Antibody | LI-COR Biosciences | Cat#926-32210  RRID: AB_621842 | 1:10000 (WB) |

CUT&Tag, Cleavage Under Targets and Tagmentation; IHC, immunohistochemistry; IP, immunoprecipitation; mAb, monoclonal antibody; pAb, polyclonal antibody; WB, western blot

**Table S3. Primers for RT-qPCR used in this study**

| **Gene Symbol** | **Gene Name** | **Gene ID** | **Primer Sequences（5'-3'）** |
| --- | --- | --- | --- |
| *TTK* | TTK protein kinase | 7272 | F: TGGCCAACCTGCCTGTTT  R: AATGCATTCATTTGCTGAAGAAGA |
| *BUB1B* | BUB1 mitotic checkpoint serine/threonine kinase B | 701 | F: AAATGACCCTCTGGATGTTTGG  R: GCATAAACGCCCTAATTTAAGCC |
| *ACTB* | actin beta | 60 | F: CATGTACGTTGCTATCCAGGC  R: CTCCTTAATGTCACGCACGAT |

**Table S4. Primers for ChIP-qPCR used in this study**

| **Gene Symbol** | **Gene Name** | **Gene ID** | **Primer Sequences（5'-3'）** |
| --- | --- | --- | --- |
| *TTK* | TTK protein kinase | 7272 | F: CGGTTACTCCTCGTCTGC  R: CAACCCGTCCTACCACAG |
| *BUB1B* | BUB1 mitotic checkpoint serine/threonine kinase B | 701 | F: ATTCAGGGCTTTGATTGT  R: TGTGGAGGACTTTGATGTA |

**Table S5. Kits and reagents used in this study**

| **Kit/Reagent** | **Source** | **Identifier (Cat#)** |
| --- | --- | --- |
| Lactic Acid Content Assay Kit | Solarbio Life Sciences | BC2235 |
| Lactate Dehydrogenase Assay Kit | Nanjing Jiancheng Bioengineeering Institute | A020-2-2 |
| MinElute PCR Purification Kit | Qiagen | 28004 |
| DCA (Sodium Dichloroacetate) | Sigma | 347795 |
| Oxamate (Sodium Oxamate) | MedChemExpress | HY-W013032A |
| 2-DG (2-Deoxy-D-glucose) | MedChemExpress | HY-13966 |
| NaLa (Sodium Lactate) | Sigma | 1614308 |
| C646 | Selleck | S7152 |
| TSA (Trichostatin A) | Selleck | S1045 |
| NAM (Nicotinamide) | Selleck | S1899 |
| CI994 (Tacedinaline) | Selleck | S2818 |
| TMP195 | Selleck | S8502 |
| Bufexamac | MedChemExpress | HY-B0494 |
| SIS17 | Selleck | S6687 |
| BeyoMag™ Protein A+G Magnetic Beads | Beyotime | P2108 |
| Dynabeads™ Protein G | Invitrogen | 10004D |

**Supplementary figures**


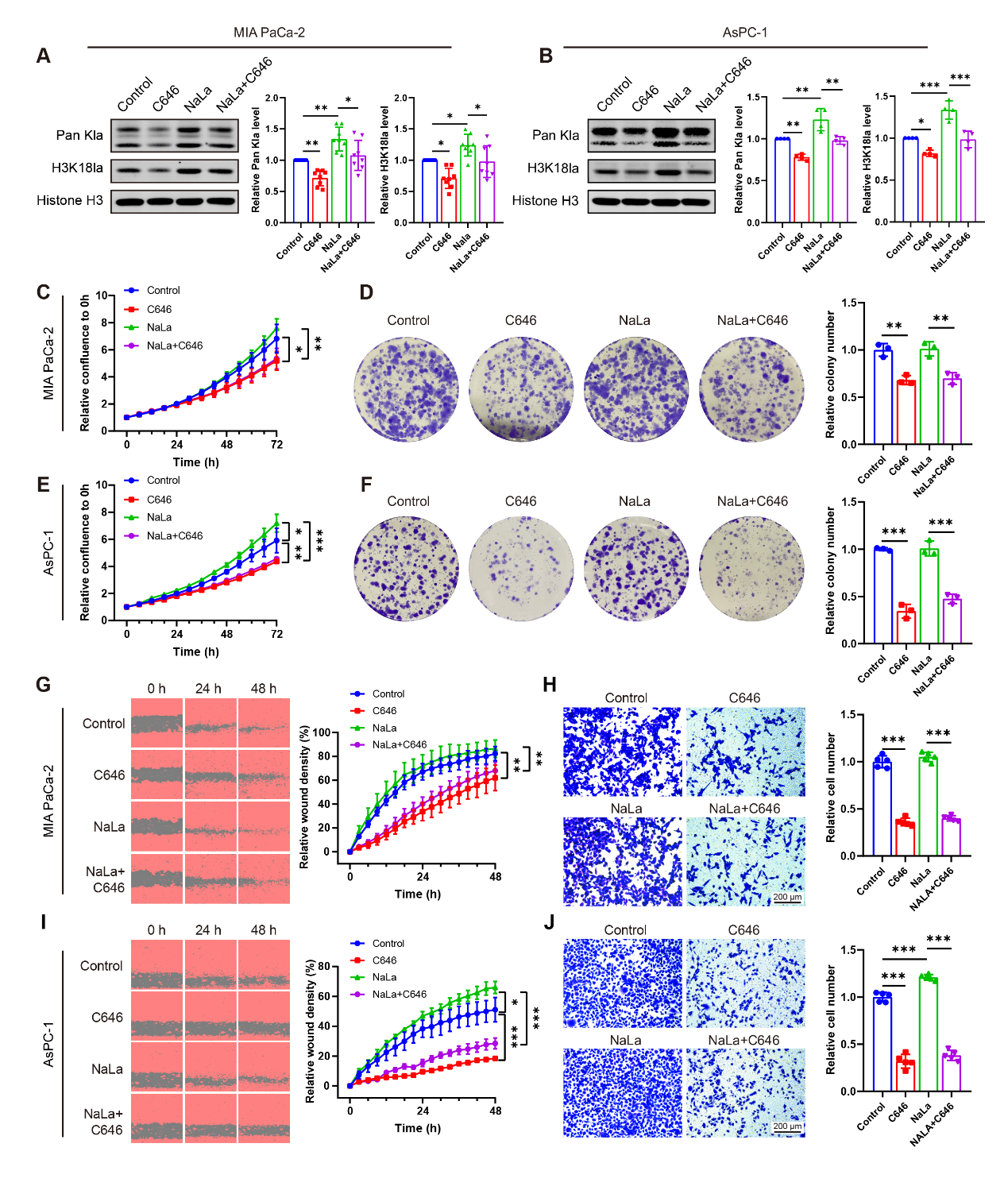


**Figure S1. P300 is a potential writer of histone lactylation in PDAC cells**

Two PDAC cell lines (MIA PaCa-2 and AsPC-1) were treated with P300 inhibitor C646 (20 μmol/L), NaLa (10 mmol/L) or NaLa combined with C646. (**A**-**B**) The pan-lysine lactylation (Pan Kla) and H3K18 lactylation (H3K18la) levels were measured by western blot and quantified using Image J software. *n* = 8 or 4. (**C**-**F**) Cell proliferation was assessed by using IncuCyte S3 (**C**, **E**) and colony formation assays (**D**, **F**). *n*=5 or 3. (**G**-**J**) The migration ability was assessed using wound healing (**G**, **I**) and transwell assays (**H**, **J**). *n* = 6, 3 or 5. All data are presented as mean ± SD. Statistical analysis was performed by ANOVA followed by Tukey’s multiple comparisons test. ^*^*P* < 0.05, ^**^*P* < 0.01, ^***^*P* < 0.001


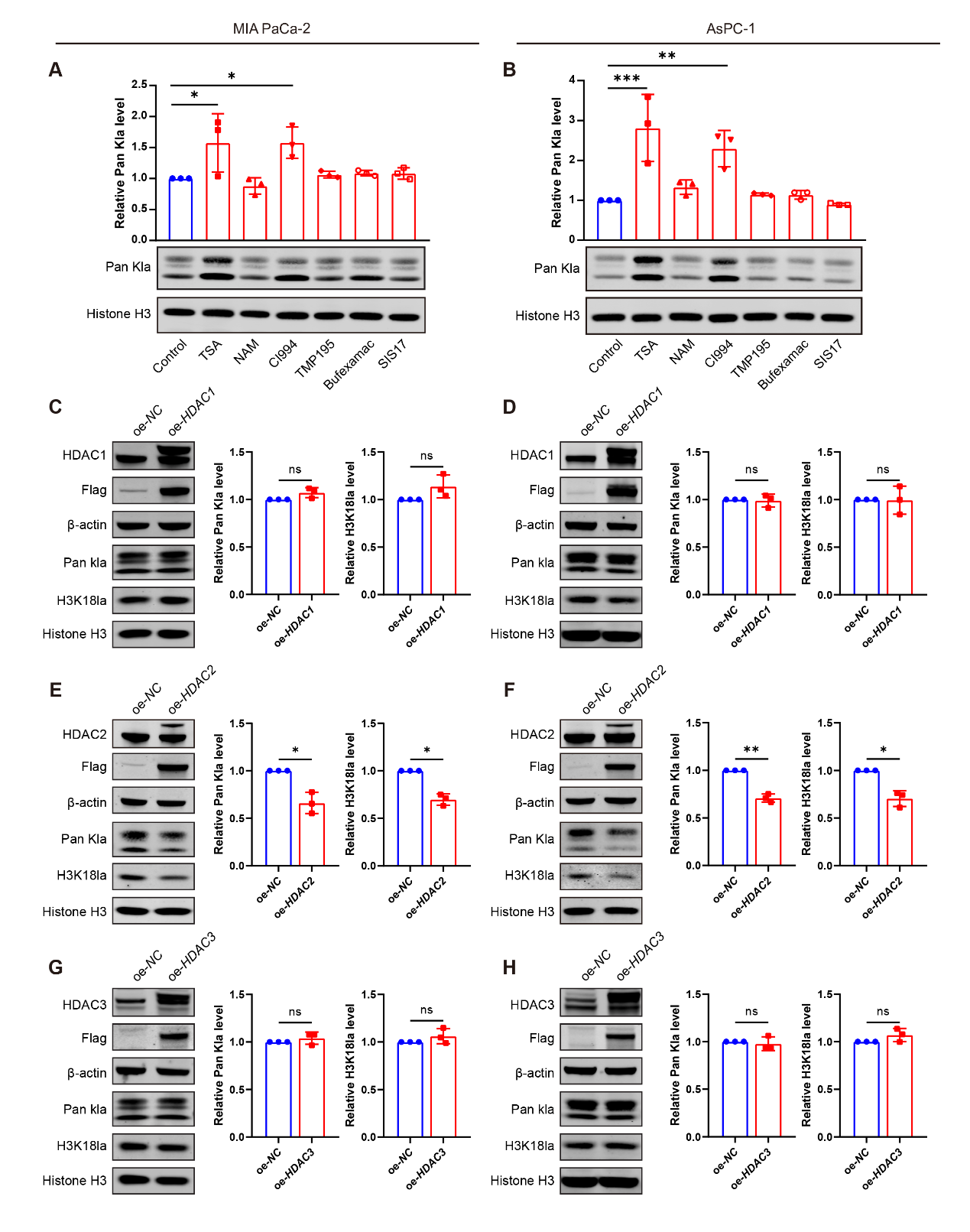


**Figure S2. HDAC2 is a potential eraser of histone lactylation in PDAC cells**

(**A**-**B**) PDAC cell lines MIA PaCa-2 (**A**) and AsPC-1 (**B**) cells were treated with various HDAC inhibitors for 24 h. The pan-lysine lactylation (Pan Kla) and H3K18 lactylation (H3K18la) were measured by western blot and quantified using Image J software. *n* = 3. TSA, pan-HDAC inhibitor, 500 nmol/L; NAM, pan-sirtuin inhibitor, 10 mmol/L; CI994, class I HDAC inhibitor, 5 μmol/L; TMP195, class IIa HDAC inhibitor, 5 μmol/L; Bufexamac, class IIb HDAC inhibitor, 250 μmol/L; SIS17, class IV HDAC inhibitor, 25 μmol/L. (**C-H**) MIA PaCa-2 (**C**, **E**, **G**) and AsPC-1 (**D**, **F**, **H**) cells were transfected with *HDAC1* (**C**, **D**), *HDAC2* (**E**, **F**) or *HDAC3* (**G**, **H**) overexpression plasmids with a *FLAG* tag for 48 h. The levels of Pan Kla and H3K18la were determined by western blot and quantified using Image J software. *n* = 3. All data are presented as mean ± SD. Statistical analysis was performed by one-way ANOVA followed by Dunnett’s multiple comparisons test in A-B, or by Student’s *t-*test in C-H. ^*^*P* < 0.05, ^**^*P* < 0.01, ^***^*P* < 0.001; ns, not significant


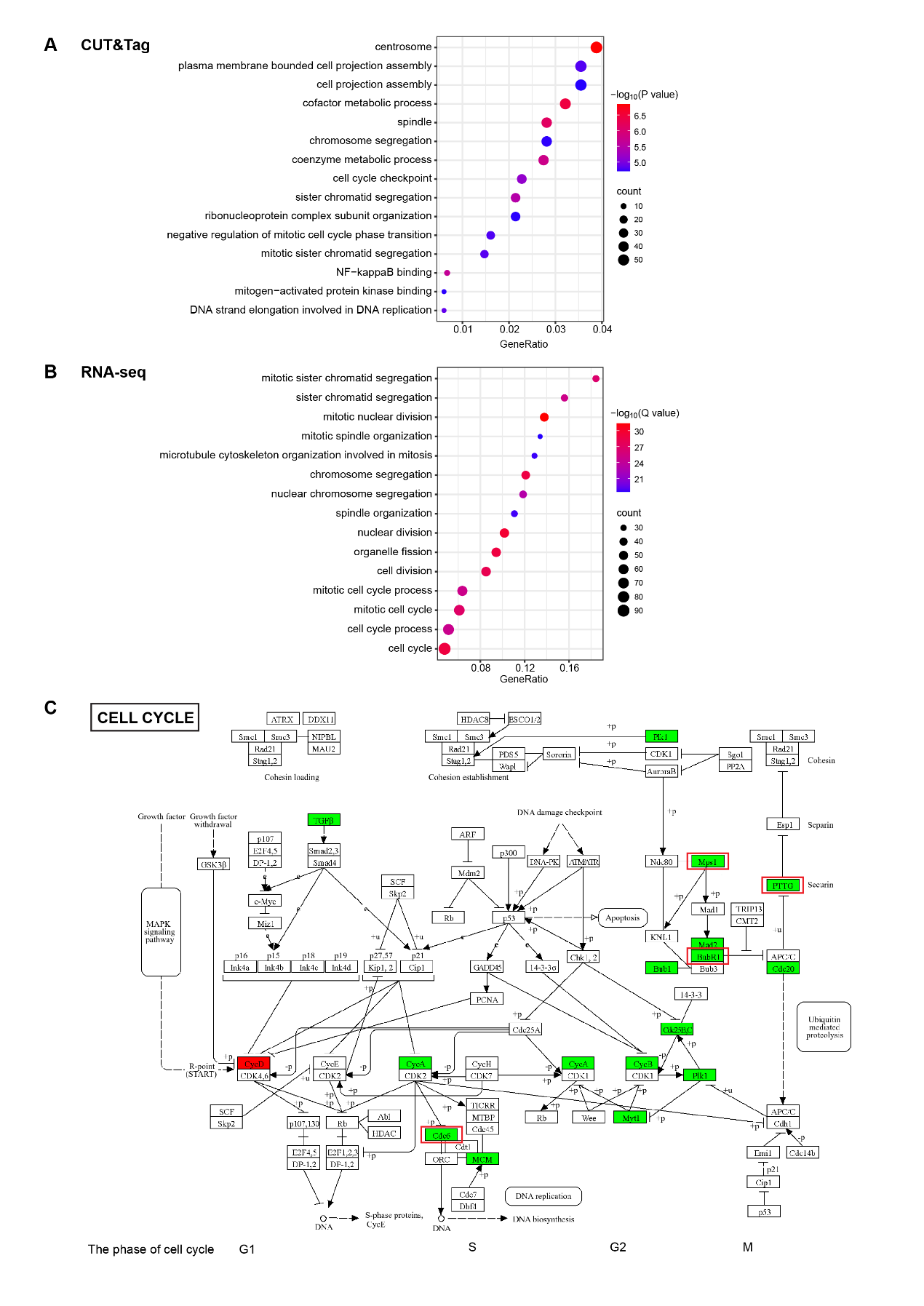


**Figure S3. Cell cycle pathway is enriched following glycolysis inhibition analyzed by GO and KEGG analysis in MIA PaCa-2 cells**

PDAC cell line MIA PaCa-2 cells were treated with LDHA inhibitor Oxamate (10 mmol/L) or vehicle for 24 h. Cells were collected for CUT&Tag by using H3K18la antibody to detect the potential binding sites, and for RNA-seq to reveal the downstream genes. (**A**) GO analysis of the loss H3K18la peaks in the promoter region in CUT&Tag assay. (**B**) GO analysis of the downregulated genes in RNA-seq. (**C**) The changes of genes related to cell cycle pathway in RNA-seq. The upregulated gene was filled in red and the downregulated genes were filled in green. The genes in the red box are the intersection genes of CUT&Tag (genes in A), RNA-seq (genes in B) and GEPIA database (genes with high levels in PDAC). Mps1, also known as TTK; BubR1, also known as BUB1B


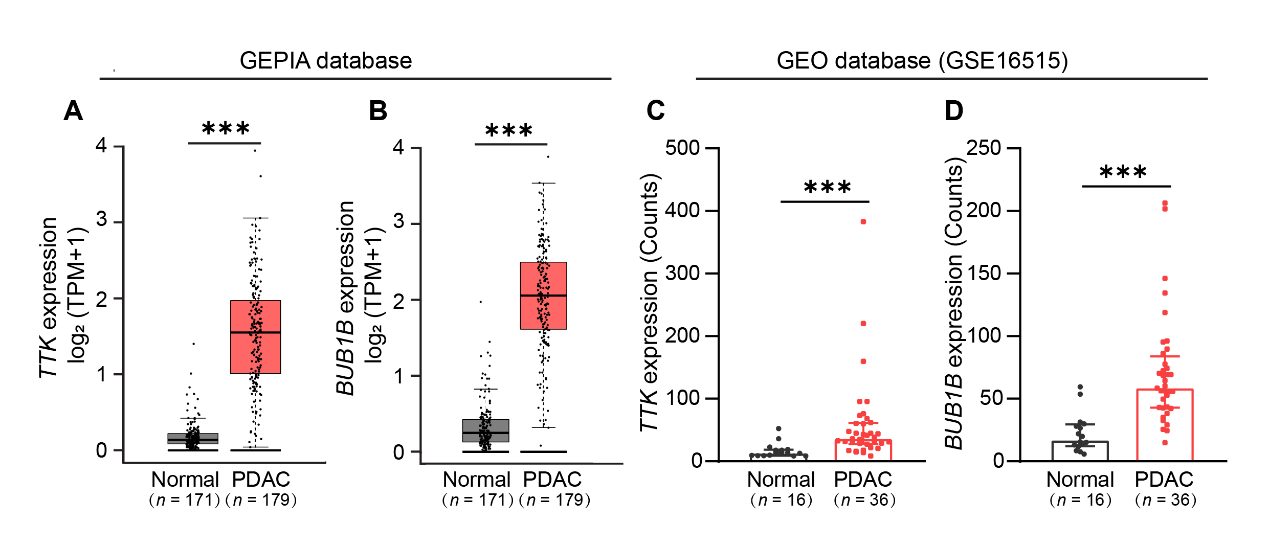


**Figure S4. TTK and BUB1B are highly expressed in PDAC**

(**A**-**B**) Expression of *TTK* (**A**) and *BUB1B* (**B**) in normal and PDAC tissues in GEPIA database. (**C**-**D**) Expression of *TTK* (**C**) and *BUB1B* (**D**) in normal and PDAC tissues in GEO database (GSE16515). All data are presented as median with interquartile range. Statistical analysis was performed by Mann-Whitney test. ^***^*P* < 0.001. TPM, transcripts per million

**
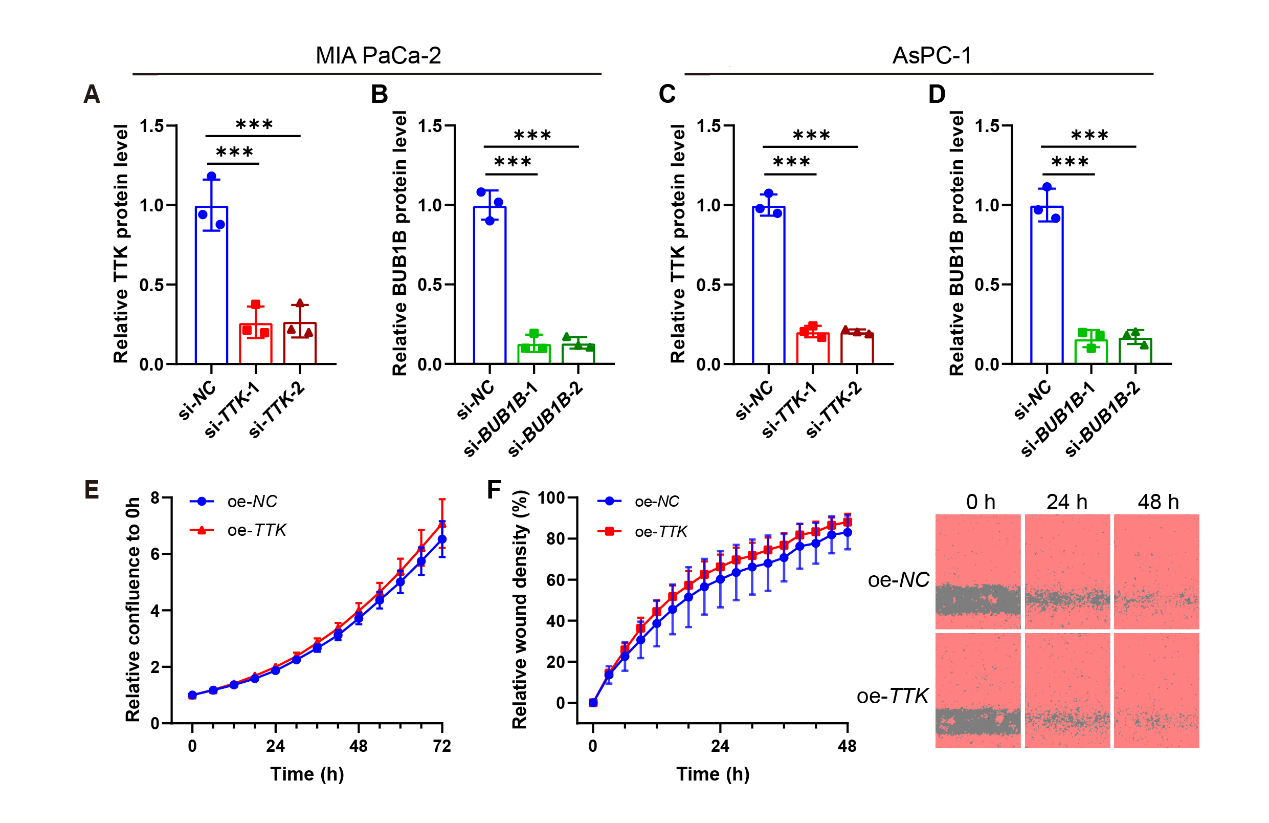
**

**Figure S5. TTK and BUB1B are successfully silenced after siRNA, and *TTK* overexpression has no effect on the proliferation and migration in PDAC cells**

(**A-D**) MIA PaCa-2 cells and AsPC-1 cells were transfected with *TTK* siRNA (si-*TTK*), *BUB1B* siRNA (si-*BUB1B*), or negative control siRNA (si-*NC*) for 48 h. Quantification of the efficiency of si-*TTK* (**A**, **C**) and si-*BUB1B* (**B**, **D**) in MIA PaCa-2 cells (**A**, **B**) and AsPC-1 cells (**C**, **D**). *n* = 3. (**E-F**) MIA PaCa-2 cells were transfected with *TTK* overexpression lentivirus (oe-*TTK*) or negative control lentivirus (oe-*NC*) for 72 or 48 h. The proliferation ability (**E**) and migration ability (**F**) were detected by using IncuCyte S3. *n* = 6 or 5. All data are presented as mean ± SD. Statistical analysis was performed by ANOVA followed by Dunnett’s multiple comparisons test in A-D, or by Student’s *t-*test in E-F. ^***^*P* < 0.001

**
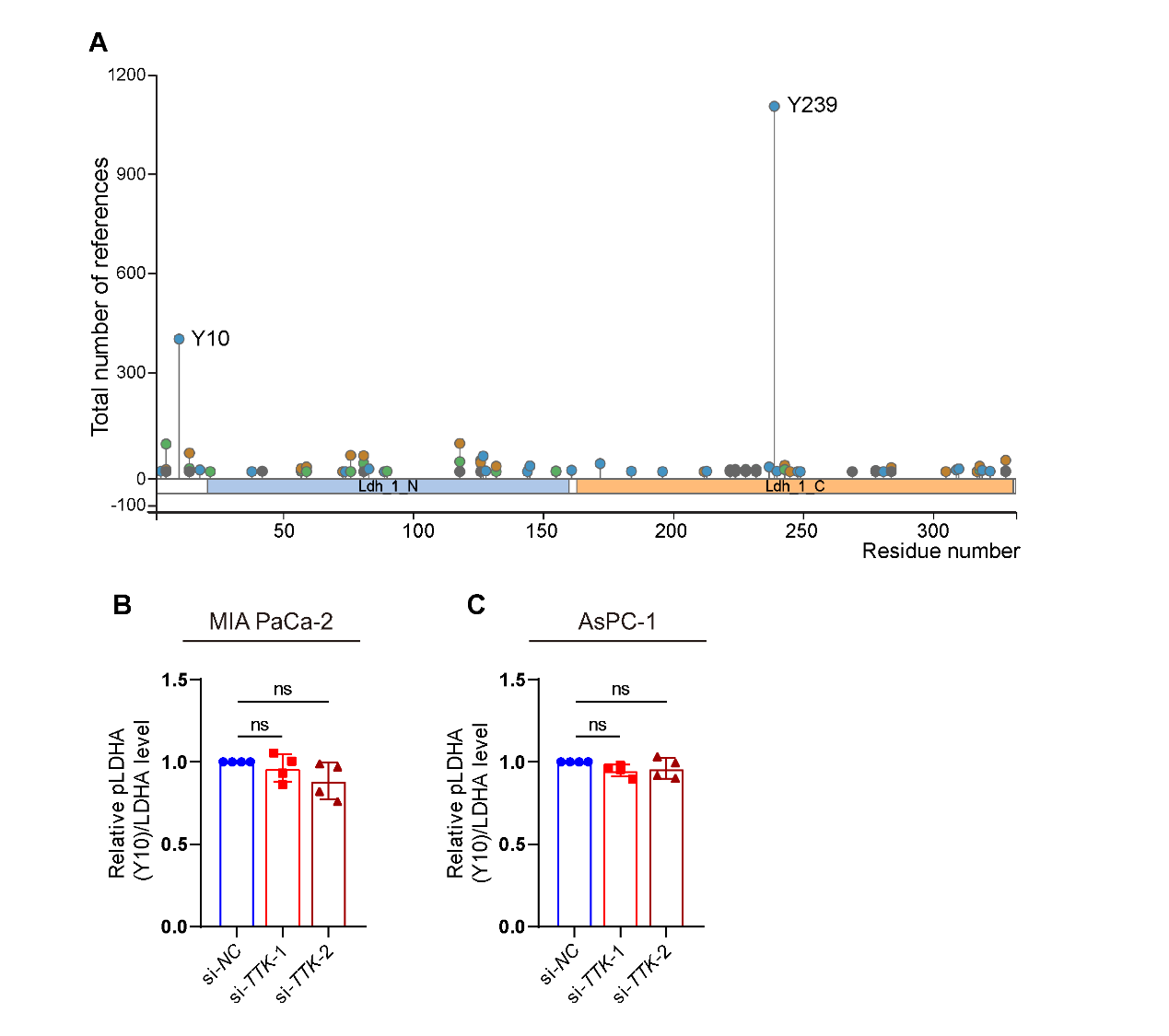
**

**Figure S6. TTK has no effect on LDHA phosphorylation at Y10 in PDAC**

(**A**) PhosphoSitePlus predicted potential phosphorylation sites on LDHA, including tyrosine 10 (Y10) and tyrosine 239 (Y239) (<https://www.phosphosite.org/homeAction>). (**B-C**) MIA PaCa-2 (**B**) and AsPC-1 (**C**) cells were transfected with negative control siRNA (si-*NC*) or *TTK* siRNA (si-*TTK*) for 72 h. The protein levels of phosphorylated LDHA at Y10 were detected by western blot and quantified. *n* = 4. All data are presented as mean ± SD. Statistical analysis was performed by one-way ANOVA followed by Dunnett’s multiple comparisons test in B and C. ns, not significant
